# New 1,2,3-Triazoles from (R)-Carvone: Synthesis, DFT Mechanistic Study and In Vitro Cytotoxic Evaluation

**DOI:** 10.3390/molecules27030769

**Published:** 2022-01-25

**Authors:** Ali Oubella, Abdoullah Bimoussa, Abdellah N’ait Oussidi, Mourad Fawzi, Aziz Auhmani, Hamid Morjani, Abdelkhalek Riahi, M’hamed Esseffar, Carol Parish, Moulay Youssef Ait Itto

**Affiliations:** 1 Laboratoire de Chimie Moléculaire, Département de Chimie, Faculté des Sciences, Semlalia B.P 2390, Marrakech 40001, Morocco; oubellaali1@gmail.com (A.O.); bimoussa_@hotmail.com (A.B.); docabdellah@gmail.com (A.N.O.); fawzi.mourad93@gmail.com (M.F.); a.auhmani@uca.ac.ma (A.A.); 2 BioSpectroscopie Translationnelle, BioSpecT—EA7506, UFR de Pharmacie, Université de Reims Champagne-Ardenne, 51 Rue Cognacq Jay, CEDEX, 51096 Reims, France; hamid.morjani@univ-reims.fr; 3 Equipe MSO, CNRS UMR 7312 Institut de Chimie Moléculaire, Université de Reims Champagne-Ardenne, Bat. Europol’Agro-Moulin de La Housse UFR Sciences B.P., 1039, CEDEX 2, 51687 Reims, France; abdelkhalek.riahi@univ-reims.fr; 4 Gottwald Science Center, 28Westhampton Way, University of Richmond, Richmond, VA 23173, USA

**Keywords:** (R)-carvone, 1,2,3-triazole, DFT calculations, regioselectivity, cytotoxic activity

## Abstract

Aseries of novel 1,4-disubstituted 1,2,3-triazoles were synthesized from an (R)-carvone terminal alkyne derivative via a Cu (I)-catalyzed azide–alkyne cycloaddition reaction using CuSO_4_,5H_2_O as the copper (II) source and sodium ascorbate as a reducing agent which reduces Cu (II) into Cu (I). All the newly synthesized 1,2,3-triazoles **9a**–**h** were fully identified on the basis of their HRMS and NMR spectral data and then evaluated for their cell growth inhibition potential by MTS assay against HT-1080 fibrosarcoma, A-549 lung carcinoma, and two breast adenocarcinoma (MCF-7 and MDA-MB-231) cell lines. Compound **9d** showed notable cytotoxic effects against the HT-1080 and MCF-7 cells with IC_50_ values of 25.77 and 27.89 µM, respectively, while compound **9c** displayed significant activity against MCF-7 cells with an IC_50_ value of 25.03 µM. Density functional calculations at the B3LYP/6-31G* level of theory were used to confirm the high reactivity of the terminal alkyne as a dipolarophile. Quantum calculations were also used to investigate the mechanism of both the uncatalyzed and copper (I)-catalyzed azide–alkyne cycloaddition reaction (CuAAC). The catalyzed reaction gives complete regioselectivity via a stepwise mechanism streamlining experimental observations. The calculated free-energy barriers 4.33 kcal/mol and 29.35 kcal/mol for the 1,4- and 1,5-regioisomers, respectively, explain the marked regioselectivity of the CuAAC reaction.

## 1. Introduction

**Scheme 1:**
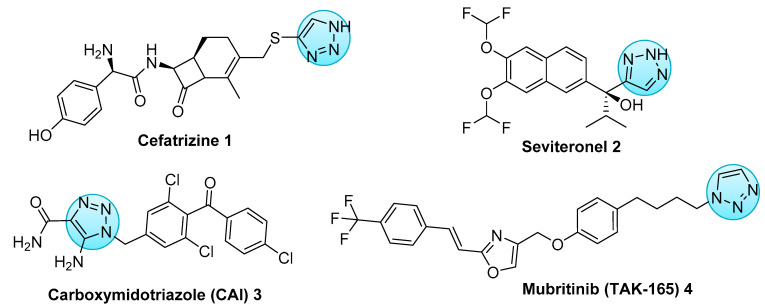
Potential anticancer drugs based on 1,2,3-triazole nucleus.

Cancer is a significant public health issue and has become the leading cause of death worldwide [1]. One of the hallmarks of cancer is the ability of some tumor cells to evolve during the epithelial mesenchymal transition and to acquire both migration and invasion properties. These two characteristics are the most important key factors in metastasis [2] and often lead to poor prognosis and treatment failure. For tumor genesis, genetic predisposal and environmental carcinogenic factors are among the leading causes of the most common forms of cancer [3]. Recent advances in therapeutic research have successfully developed new potent anticancer agents capable of targeting tumors with minimal side effects [4,5]. Natural compounds have been and are the main source for the development of several anticancer agents [6,7,8,9,10]. Terpenes and their functionalized derivatives, especially heterocyclic analogues, are often used as a source for the preparation of new semi-synthetic compounds displaying various biological properties including anticancer activity [11,12]. 1,2,3-Triazole derivatives of such terpenes have been shown to present a broad spectrum of biological properties including antiproliferative, antiretroviral, and antimicrobial activities [13,14,15,16,17,18]. Recently, nitrogen-containing heterocyclic compounds have attracted considerable attention in the field of anticancer research [19,20]. Among them, 1,2,3-triazolic systems constitute an important class of five-membered heterocyclic compounds that exhibit promising anticancer properties. Significant and interesting properties have been reported for several drugs containing the 1,2,3-triazole moiety, such as cefatrizine **1** [21] and seviteronel **2** [22]. As of 2021, some of these molecules such as carboxyamidotriazole or CAI **3** [23] and mubritinib or TAK-165 **4** [24] (Scheme 1) are in active clinical trials.

In terms of stability, 1,2,3-triazoles are highly stable under oxidative and acidic or basic hydrolysis conditions [25]. 1,2,3-Triazoles possess a significant dipole moment (~5 Debye) and have the ability to form hydrogen bonds, which facilitates binding with the biomolecular targets and improves their aqueous solubility [26,27]. 1,2,3-Triazoles are generally prepared by an azide–alkyne cycloaddition reaction. However, this synthetic procedure, first proposed by Huisgen [28], often leads to two regioisomers: 1,4- and 1,5-disubstituted 1,2,3-triazoles. It was only after the introduction of the click chemistry concept by Sharpless [29], which has developed the stereospecific synthesis of 1,4-regioisomer (in the presence of Cu (I) catalyst at room temperature), that this methodology has gained great interest in synthetic and medicinal chemistry. The efficient and regiospecific alkyne-azide-click reaction, also known as the Cu-catalyzed Alkyne-Azide Cycloaddition reaction (CuAAC), has become a common approach for the stereoselective synthesis of the 1,4 regioisomer of 1,2,3-triazoles. As a part of our efforts toward the synthesis of new bioactive heterocyclic systems with a basic terpenic skeleton [30,31,32,33,34,35,36,37], we have recently reported the interesting anticancer activities of some 1,4-disubstituted 1,2,3-triazolic compounds newly prepared from Eugenol and (R)-carvone monoterpene [38,39]. These promising findings prompt us to extend our interest in the synthesis ofother 1,4-disubstituted 1,2,3-tiazoles from another natural monoterpene. In the present work, we report the preparation of several 1,4-disubstituted 1,2,3-triazoles built on an (R)-carvone skeleton, obtained chemospecifically via the regiospecific (CuAAC) reaction of arylazides on a terminal alkyne prepared from natural (R)-carvone. A DFT theoretical study was carried out to account for the peri- and the regiospecificity of the reaction. We have also evaluated their vitro cytotoxic effects of the newly prepared triazolic compounds against four human tumors: HT-1080 fibro sarcoma, A-549 lung carcinoma, and two breast adenocarcinoma (MCF-7 and MDA-MB-231) cell lines.

## 2. Result and Discussion

### 2.1. Chemistry

**Scheme 2:**
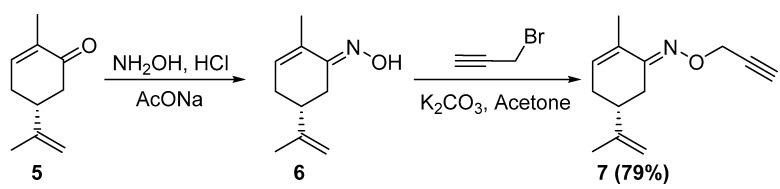
Synthetic route for the preparation of terminal alkyne **7**.

The terminal alkyne **7** is the required precursor for the synthesis of the target 1,2,3-triazoles. It was synthesized according to the reported two-step procedure [40], starting from (R)-carvone **5,** which was first transformed into the corresponding oxime **6** (Scheme 2). In the second step, the oxime **6** was alkylated with propargyl bromide in dry acetone, using threeequivalents of potassium carbonate as base. After 5 h of reactionat room temperature, then purification by liquid chromatography on a silica gel column, we obtained the corresponding terminal alkyne **7** in 79% yield (Scheme 2). The structure of **7** was fully identified from its HRMS and NMR spectral data which were in full accordance with the reported ones [40]. Its HRMS spectra showed the corresponding pseudo-molecular ion [MH]^+^ at *m*/*z* = 204.1388 is consistent with its molecular formula C_13_H_17_NO. Its ^1^H NMR spectrum exhibited characteristic signals such as two one proton triplets (*J* ≈ 5 Hz) at δ 4.62 and δ 4.69 due to O-CH_2_ and atriplet (*J* ≈ 5 Hz) at δ 2.38 due to theacetylenic proton. The same propargyloxy group is revealed in the ^13^C NMR spectrum by the resonance of the methylenic and acetylenic carbons at δ 62.93, 74.37 and 80.20 ppm.

In the subsequent step, the terpenic terminal alkyne **7** was submitted to the Cu (I)-catalyzed azide–alkyne cycloaddition reaction (CuAAC) also named the azide–alkyne-click reaction, which is renowned for its high efficiency and regiospecificity when producing 1,4-disubstituted 1,2,3-triazoles. The reaction was carried out for 6 h at room temperature, with aromatic azides **8a**–**h**, using 15 mol% of CuSO_4_ as the copper source, in the presence of sodium ascorbate (20 mol%) acting as a Cu^2+^ reductant, in EtOH-H_2_O (1/5) mixture as the solvent [41].

**Scheme 3:**
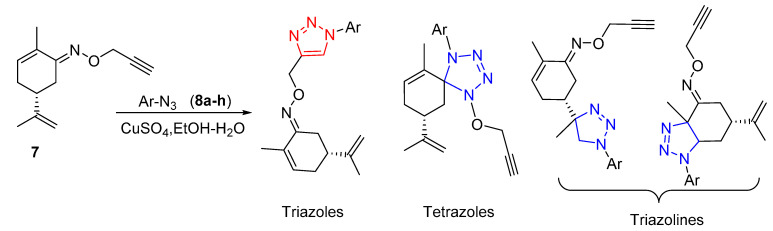
The obtainable heterocyclic systems from CuAAC reaction of **7** with aromatic azides **8a**–**h**.

It should be emphasized that as compound **7** features other dipolarophile moieties such as C=N and the two C=C double bonds, the cycloaddition reaction could lead to other heteocyclic systems such astetrazoles [42] and/or triazolines [43] (Scheme 3).

**Scheme 4:**
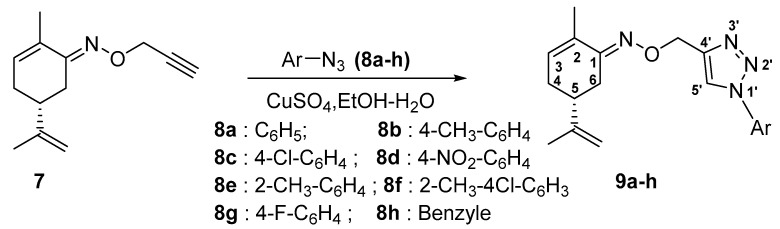
General schematic for the synthesis of novel 1-4-disubstituted 1,2,3-triazoles **9a**–**h**.

After the work up anda silica gel chromatography purification of the reaction mixture, we isolated the corresponding cycloadducts **9a**–**h** in good yields (Scheme 4, Table 1).

All the new terpenic1,2,3-triazoles **9a**–**h** were identified by HRMS and NMR spectroscopic techniques. Indeed, their ESI-HRMS spectra reveal pseudo-molecular ions [MH]^+^ consistent with the corresponding molecular formula (Table 1).

In their NMR spectra, which show a close similarity, the main feature were the disappearance of the starting acetylenic moiety resonances (δ ^1^H2.38 ppm; δ ^13^C 74.37 and 80.20 ppm), while we still note signals of both =C3H (δ ^1^H 5.96 ppm; δ ^13^C132.82 ppm) and =CH_2_ of the carvone core (δ ^1^H 4.76 ppm; δ ^13^C109.96 ppm) (Table 2). These data prove unambiguously that the cycloaddition of the azides **8a**–**h** has occurred perispecifically on the acetylenic dipolarophile, leading to the 1,2,3-triazole ring formation. Thelatter is revealed in **9a**–**h** ^1^H NMR spectra by a one proton singlet rangingfrom 7.50 to 8.35 ppm due to the soletriazolic proton and in ^13^C NMR spectra by the C4′ and C5′ carbons resonances noticed, respectively, at around145.93 and 121.74 ppm (Table 2).

**Figure 1:**
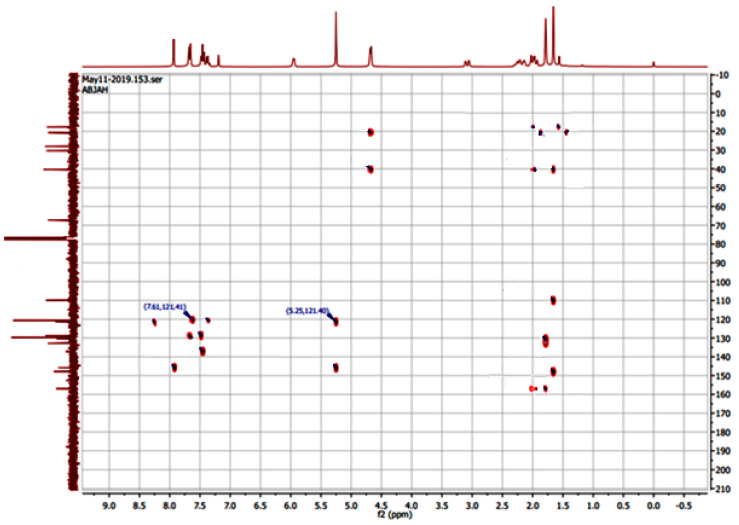
The HMBC correlations of **9a**.

In addition to the newly revealed protons and carbons of the triazolic N-aryl groups, the NMR spectra of **9a**–**h** exhibit the methylenoxy group O-CH_2_ as a two-proton singlet (δ ^1^H 5.54–5.26) and an isolated ^13^C signal in the region 66.98–67.29 ppm. To confirm the well-established regiospecificity of the CuAAC reaction for the 1,4-disubstituted 1,2,3-trizoles [29], we have examined the 2D NMR HMBC (**H**eteronuclear **M**ultiple **B**ond **C**orrelation) spectrum of **9a** (Figure 1), which shows correlations (*J4* coupling) between the C5′ carbon (δ ^13^C 121.4 ppm) and protons of the N-phenyl group (δ ^1^H 7,61 ppm). All spectral data of the synthesized products are given in the Supporting Experimental Information. 

In an attempt to clarify the high periselectivity and regioselectivity of the CuAAC reaction of arylazides **8a**–**h** with the carvonic terminal alkyne **7**, we have carried out a theoretical study using density functional theory (DFT).

### 2.2. Theoretical Results and Discussion

#### 2.2.1. Analysis of the DFT Reactivity Indices

It is known from several studies that global indices [44,45] defined in the context of density functional theory (DFT) [46,47] are very useful tools to understand the behavior of polar cycloadditions [44,45]. In Table 3 are presented the static global properties of the reagents, namely the electronic chemical potential (µ, chemical hardness (η), global electrophilicity (ω, and global nucleophilicity (N).

The chemical potentials of **8a** and **7** are very close, (−3.62 and −3.34 eV, respectively), indicating that the reaction will have a non-polar character. This behavior is confirmed by the low values of GEDT computed at the corresponding TSs. The values of Table 3 indicate that arylazide **8a** is a strong electrophile, ω = 1.27 eV and a moderate nucleophile, N = 2.92 eV, within the electrophilicity [48] and nucleophilicity [49] scales. The acetylene carvone derivative **7** is a marginal electrophile, ω = 1.07 eV, and it is just ontheborder between moderate and strong nucleophiles, N = 3.17 eV. The nucleophilicity difference between the two reagents is relatively low (∆N = 0.25 eV), suggesting a lack of polar character inthe reaction. However, the coordination of the binuclear Cu(I) to the acetylene carvone derivative increases its chemical potential µ almost twice (from −3.34 to −6.21 eV). Its electrophilicity also increases from 1.07 to 4.42 eV, indicating that coordination converts it to a strong electrophile. On the other hand, its nucleophilicity decreases to 0.73 eV (a marginal nucleophile). This result indicates that the Cu(I) complex will react as a strong electrophile in the reaction with a strongpolar character.

#### 2.2.2. Uncatalyzed Reaction of **7** and **8a**

**Scheme 5:**
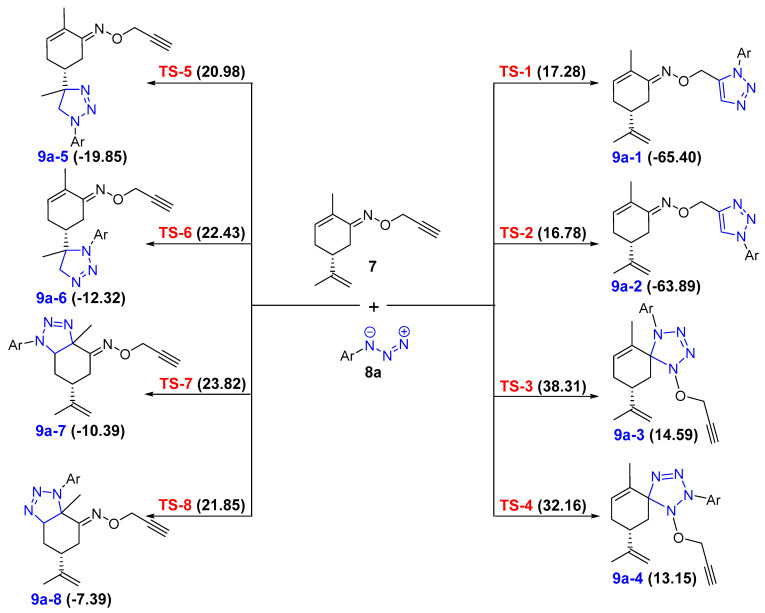
Activation energies in terms of Gibbs free energies (kcal/mol) of all the transition states associated with the corresponding regioisomers of the uncatalyzed 32CA reaction of **7** and **8a**. The energy reference is the separated reagents(values in kcal/mol).

As mentioned above, we have carried out a study at the B3LYP/6-31G* level of the uncatalyzed 32CA reaction of **7** and **8a**. The raw results are presented in Table S1 of the Calculated Supporting Information. As **7** presents four possible dipolarophile sites, the addition of **8a** can take place on each dipolarophile according to the two possible directions, leading to eight regioisomers (Scheme 5). To this end, we have studied all the possible reaction pathways. The analysis of the results for these reactions indicates that they take place along concerted bond formation processes. There are eight TSs, **TS1**–**TS8**, associated with the corresponding regioisomers **9a-1** to **9a-8**, resulting from the addition of **7** on to **8a**. All TSs and cycloadducts have been located and characterized. The relative energies, enthalpies and Gibbs energies are summarized in Table S2 of the Calculated Supporting Information. The activation energies in terms of∆G are depicted in Scheme 5.

**Scheme 6:**
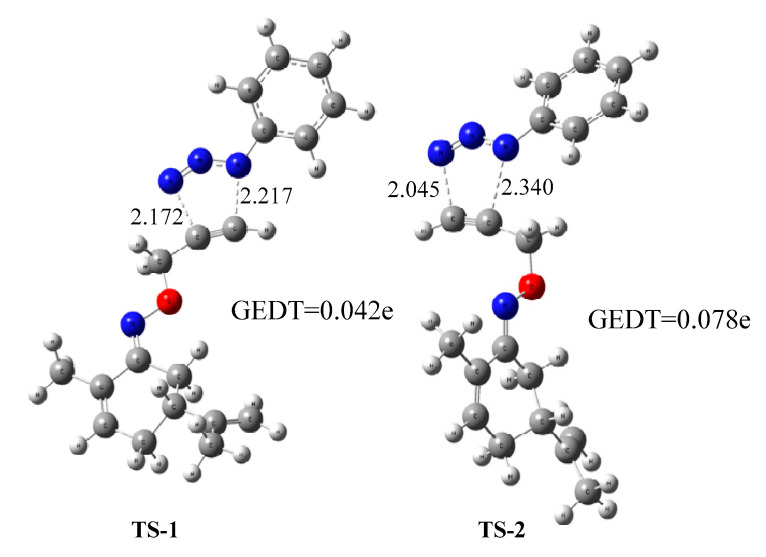
Optimized geometries of the regioisomeric**TS-1** and **TS-2** associated with the uncatalyzed 1,3-dipolar cycloaddition reaction of **7** and **8a**. The distances are given in Å.

These results show that the reaction takes place with total chemoselectivity. Only the acetylene moiety participates as a dipolarophile to yield the formation of **9a-1** and **9a-2** as the most favorable cycloadducts. This confirms the high periselectivity of the cycloaddition reaction of **7** with arylazide **8a**.The energy barriers associated with the regioisomeric pathways are not very high (ranging between 16.78 and 38.31 kcal/mol). The TSs associated with the formation of **9a-1** and **9a-2**, (**TS-1** and **TS-2**) are 17.28 and 16.78 kcal/mol, respectively, which are significantly lower than those associated with the formation of the other regioisomers. The corresponding cycloadducts, **9a-1** and **9a-2**, are energetically the most exothermic ones (−65.40 and −63.89 kcal/mol). Therefore, these two regioisomers are the kinetic and thermodynamic cycloadducts of the 1,3-dipolar cycloaddition reaction of **7** and **8a**. However, the free-energy differences (∆G) between **TS-1** and **TS-2** (0.5 kcal/mol), and between **9a-1** and **9a-2** (1.51 kcal/mol) are small, explaining the lack of regioselectivity when the 1,3-dipolar cycloaddition reaction of **7** and **8a** is carried out without any catalyst. The geometries of the **TS-1** and **TS-2** are given in Scheme 6. The lengths of the C-N forming bonds are 2.172 and 2.217Å in the case of **TS-1** and 2.045 and 2.340 Å in the case of **TS-2**.

As can be seen in Scheme 6, the C-N bonds form more synchronously in TS-1 than in TS-2. The degree of bond formation asynchrony can be measured via the difference between the two forming bond lengths, 0.045Å in **TS-1** and 0.295Å in **TS-2**. This result indicates that the 1,4-regioisomer process is more asynchronous than the 1,5-regioisomer. In order to analyze the polar or non-polar character of the 32CA reaction between **7** and **8a**, the GEDT at the TSs was calculated. The values are reported in Scheme 6. The natural charges at the TSs appear to be shared between the acetylenic derivative **7** and arylazide **8a**. The GEDT, which fluxes from alkyne to azide at the TSs, is 0.042e at **TS-1** and 0.078e at **TS-2**. These low values indicate that the corresponding TSs have non-polar characters.

#### 2.2.3. Cu(I)-Catalyzed Reaction of **7** and **8a**

**Scheme 7:**
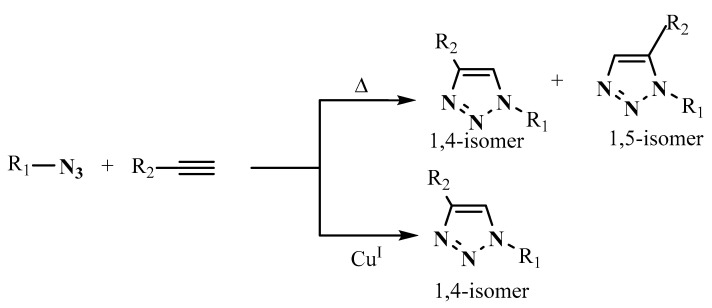
Thermaland copper-catalyzed cycloaddition reaction of azides and alkynes.

It is generally known that thermal the 1,3-dipolar cycloaddition of alkynes to azides is not a regiospecific reaction [50]. This can be very advantageous if both regioisomers are desired. Otherwise, it becomes an inconvenience if only one regioisomer is preferred. Analogous reactions have shown that the catalysis is generally regioselective (Scheme 7) [51,52,53,54,55,56,57]. The most used catalyst in this type of reaction, which is not expensive, is Cu(I). The polarization of the terminal triple bond by the covalently bound Cu(I) catalyzes the 1,3-dipolar cycloaddition, which changes from a concerted reaction into a stepwise addition [58].

**Figure 2:**
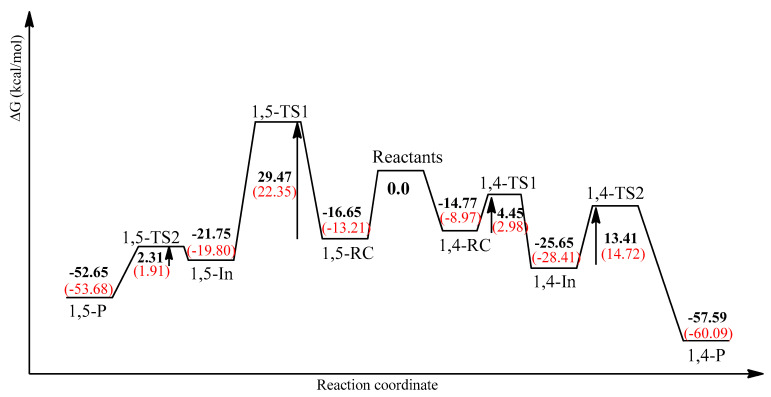
Mechanistic profiles of the regioisomeric approaches of **8a** and **2Cu(I)-7**. (Bold numbers for gas phase and numbers between brackets for ethanol as reaction medium).

The first proposal for this type of catalyzed reaction was provided by Sharpless and co-workers [52]. Cu(I) catalyzed the cycloaddition of alkynes and azides (CuAAC) constitute the entry point of click chemistry, which allowed the rapid and regiospecific synthesis of 1,4-disubstituted triazoles, largely overcoming the slowness and the non-regioselectivity of the uncatalyzed reactions according to the classical formalism. The corresponding mechanism was clarified shortly after by Finn et al. [53], who show the participation in the catalytic cycle of a second copper atom, a priori playing a role in the activation of azide functionality. Meanwhile, Straub et al. [59] succeeded in isolating a Cu(I)–triazolide complex, an intermediary of the CuAAC reaction, showing that this reaction does not necessarily imply the presence of a bi-nuclear copper complex [59]. However, other studies have shown that the reaction path with a binuclear copper complex has a lower energy barrier than a mononuclear onewhose activation energy has been found to be in the order of the uncatalyzed path [56,57]. For our part and in the same way, we only considered the catalyzed reaction path of the 1,3-dipolar cycloaddition of **7** and **8a**, involving binuclear coppercomplex. The calculated energies, zero-point vibrational energies (ZPE), thermal corrections, and entropy values of the relevant species are summarized in Table S3 of the Calculated Supporting Information. Table 4 lists their relative energies, ∆E, enthalpies, ∆H, and Gibbs energies, ∆G, relative to the reactants. The reaction involves the initial formation of a reactive complex, **RC**, which is more stable than the separate reactants. This **RC** is built on a π-coordination of the copper atom to the alkyne promoting his deprotonation and the coordination to the second copper atom. After that, the terminal nitrogen of the azide in **1,4-RC** (**1,5-RC**) binds to the C-2 carbon of the acetylide, forming the six-membered intermediate, **1,4-In** (**1,5-In**) via a first transition state **1,4-TS1** (**1,5-TS1**) whose activation barrier, in terms of ∆G, is 4.45 kcal/mol (29.47 kcal/mol) (Figure 2). This step appears to be the key mechanistic process of this reaction, allowing the formation of the 1,4-regioisomer (**1,4-P)**. The very low activation barrier (4.45 kcal/mol) leading to 1,4-disubstitued triazole regioisomer explains the enormous acceleration in the rate of this copper-catalyzed process which is considerably lower than that of the uncatalyzed reaction (about 17 kcal/mol). From the intermediates, the second step leads to the products via another transition state, **1,4-TS2** (**1,5-TS2**), with an activation barrier of 13.41 kcal/mol (2.31 kcal/mol). This mechanistic behavior is similar to other results which have proven the binuclear nature of the CuAAC mechanism [60]. It is noteworthy that the high exothermic character of the 1,4-disubstitued triazole (−57.59 kcal/mol) makes it irreversible and the low activation barrier, (4.45 kcal/mol), which is very easy to overcome, makes it the most kinetically favorable product. When usingethanol as solvent, all stationary points corresponding to the two regioisomers 1,4- and 1,5-disubstituted triazole are moderately changed (see Figure 2 and Table S4 of the Calculated Supplementing Information). In the case of **1,4-TS1** (**1,5-TS1**), which represents the key of the reaction mechanism, the activation barrier is stabilized from 4.35 to 2.98 kcal/mol (29.47 to 22.35 kcal/mol). Consequently, the formation of the most favorable 1,4-regioisomer is subject to a thermodynamic and kinetic control.

The computed energetic profiles of the reaction are represented in Figure 2, clearly showing the energetically favorable path leading to the 1,4-regioisomer.The path leading to the 1,5-regioisomer is confronted to the large activation barrier (29.47 kcal/mol) which is more than six times greater than that leading to the 1,4-regioisomer (4.45 kcal/mol).

**Scheme 8:**
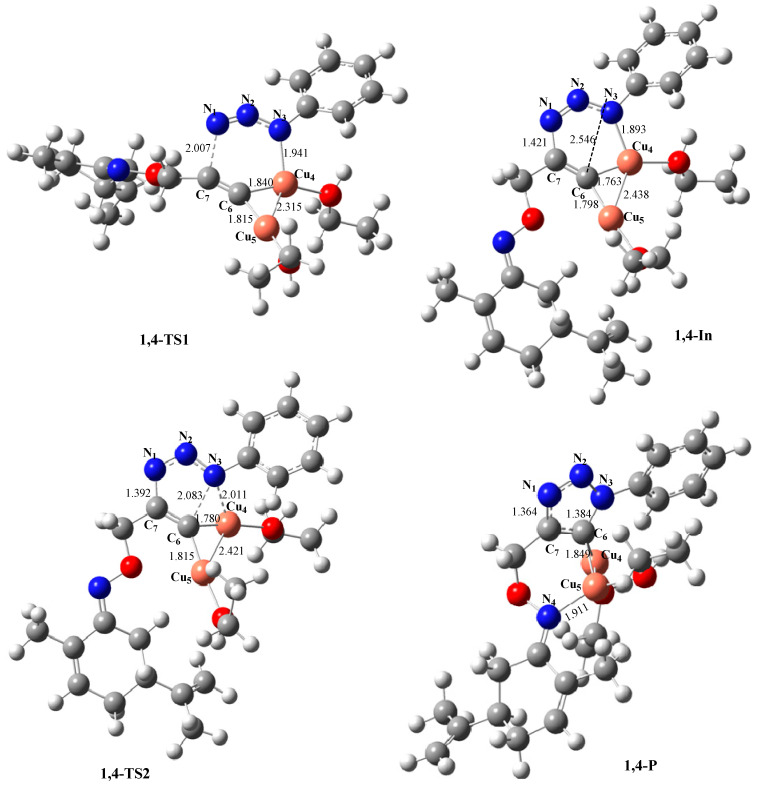
Optimized geometries of the stationary points in the 2Cu(I) catalyzed 32CA reaction. The lengths are given in Å.

The optimized structures of the stationary points of the reaction paths are shown in Scheme 8. The lengths of the two forming bonds N_1_-C_7_ and N_3_-Cu_4_, at **1,4-TS1**, are 2.007Å and 1.941Å, respectively. These two bonds evolve into the closure of the six-membered ring with N_1_-C_7_ = 1.421 Å and N_3_-Cu_4_ = 1.893 Å, showing that the formation of the reaction intermediate is much more stable than the reactive complex. At **1,4-TS2,** associated with the process of opening the six-membered metallacycle and the formation of the five-membered triazolide ring, the lengths of the N_3_-C_6_ and Cu_4_-C_6_ are 2.083 Å and 1.780 Å, respectively showing that Cu_4_ interacts strongly with both C_6_ (1.780 Å) and N_3_ (2.011 Å). The distance between C_6_ and N_3_ evolved from 2.546 Å in the intermediate to 2.083 Å, indicating the beginning of interaction of the two centers to form the C_6_-N_3_ bond with 1.384 Å at **1,4-P**. We also noted a strong interaction between Cu_5_ and nitrogen N_4_ (Cu_5_-N_4_ = 1.9 Å), inducing the formation of a six-membered ring and giving a high stability to the 1,4-regioisomer and consequently a great exothermicity.

### 2.3. Anticancer Activity

(R)-carvone oxime O-propargyl ether **7** and the monoterpenic1,2,3-triazoles **9a**–**h** were screened for their cell growth inhibitory effects, usingan MTS assay in four human cancer cell lines HT-1080 (fibrosarcoma), A549 (lung carcinoma) and two breast carcinomas (MCF-7 and MDAMB-231) [61,62,63]. The topoisomerase II inhibitor doxorubicin (DOX) was used as a positive control. Data relative to the inhibitory effect (IC_50_) of the synthesized products (**7** and **9a**–**h**) are presented in Table 5.

As shown in Table 5, the compounds **9a**, **9b, 9e** and **9f** were the less active compounds, with IC_50_ values ranging from 51.90 to 100 µM. Some of the others were able to induce moderate cell growth inhibitory effects in the different cell lines with IC_50_ values ranging from 37.50 to 48.69 µM. However, **9c** was highly active against MCF-7 cells with an IC_50_ value of 25.03 µM. The compound **9d** was more active towards HT-1080, A-549 and MCF-7 cells with respective IC_50_ values of 25.77 and 30.61 and 27.89 µM, while **9g** showed a significant cell growth inhibitory effect on HT-1080 and MCF-7 cells asits corresponding IC_50_ values were 30.44 and 30.11 µM, respectively.

## 3. Conclusions

A series of new 1,2,3-triazoles **9a**–**h**, built on (R)-carvone skeleton were synthesized, using a Cu(I)-catalyzed 1,3-dipolar cycloaddition reaction between arylazides**8a**–**h** and the monoterpenic terminal alkyne **7**. The [3+2] cycloaddition reaction was revealed to occur efficiently, with high perielectivity and regioselectivity on the carbon-carbon triple bond of **7**, affording the corresponding 1,4-disubstituted 1,2,3-triazoles. All the newly synthesized 1,4-disubstituted 1,2,3-triazoles,**9a**–**h**, were fully characterized using HRMS and NMR (^1^H and ^13^C) spectroscopic techniques. Furthermore, the high chemoselectivity of this CuAAC reaction was examined by means of DFT mechanistic studies which show that the terminal alkyne is the most reactive of all the dipolarophiles of **7**. In the absence of the catalyst, the studied reaction takes place via a concerted mechanism involving the two energetically very close regioisomers. Meanwhile, the reaction catalyzed by binuclear copper (I) gave access to the only 1,4-substituted 1,2,3-triazole regioisomer via a stepwise mechanism with a very low activation barrier, 4.45 kcal/mol (2.98 kcal/mol in ethanol), rationalizing the experimental observation. On the other hand, all the newly prepared 1,4-disubstituted 1,2,3-triazoles **9a**–**h** were evaluated for their anticancer activitiesagainst four selected human cancer cell lines. The tested compounds showed low to good cytotoxic activity against the four investigated cell lines. The compounds **9c**, **9d** and **9g** showed the most interesting cell growth inhibitory effects.

## 4. Experimental Section

### 4.1. Materials and Methods

All chemicals were used as obtained from commercial sources (Aldrich and Acros). Melting points (m.p) were determined using a capillary apparatus and are uncorrected. Analytical thin-layer chromatography (TLC) was performed on plates precoated with E. Merck silica gel 60 F254 to a thickness of 0.25 mm. HRMS were obtained on a Q-TOF micro mass spectrometer. ^1^H and ^13^C NMR spectra were recorded in CDCl_3_ with 500 MHz Bruker Advance Neospectrometer with an Iprobe. Chemical shifts (δ) are expressed in parts per million (ppm). They were recorded relative to solvent CDCl_3_ signal (7.26 ppm and 77.16 ppm). The (R)-carvoneoxime6 was prepared according to the reported method [40]. The azide derivatives were prepared from the corresponding aniline precursors according to the reported procedures [64].

### 4.2. Computational Treatment: Calculations

Quantum chemistry calculations were carried out using the Gausssian 09 program [65]. Exploration of the potential energy surface (PES) was carried out using the B3LYP functional [66,67] together with the 6-31G(d,p) basis set [68]. Optimizations were carried out using the Berny analytical gradient optimization method [69,70]. The stationary points were characterized by frequency computations in order to verify that transition states (TSs) have one and only one imaginary frequency. The IRC paths [71] were traced in order to confirm the energy profiles connecting each TS to the two associated minima of the proposed mechanism using the second order González–Schlegel integration method [72,73]. Values of enthalpies, entropies and free energies were obtained by frequency calculations over B3LYP/6-31G gas-phase geometries. Thermodynamic data were calculated with the standard statistical thermodynamics at 298.15 K and 1 atm [68]. The harmonic vibrational frequencies were determined at the same level to confirm that the optimized structures correspond to real minima of the potential energy surface and to evaluate the zero-point energy (ZPE) corrections, thermal corrections, and entropy values. The ZPE corrections were scaled by anempirical factor of 0.9806 [74]. The global electron density transfer (GEDT) [75] was calculated using the equation GEDT = Σq, where q was obtained by a Natural Population Analysis (NPA) [76,77]. As our experimental reactions were carried out in ethanol, we explored the effect of solvent the mechanistic study using ethanol as ligand interacting with the copper atom. The conceptual DFT reactivity indices [46] of the reagents were calculated as follows. The global electrophilicity [78] ω is given by the expression ω = (µ^2^/2η) in terms of the electronic chemical potential µ and the chemical hardness η [79] with µ = (E_H_ + E_L_)/2 and η = (E_L_ + E_H_). E_H_ and E_L_ are the HOMO and LUMO energies, respectively. The nucleophilicity N is obtained by N = E_H_(Nu) − E_H_(TCE). Nucleophilicity is referenced to tetracyanoethylene (TCE) because it presents the lowest HOMO energy in a long series of molecules already investigated in the context of polar cycloadditions [59]. Solvation energies in ethanol as solvent were added as single point calculations using the Polarizable Continuum Model (PCM) [80].

### 4.3. Cell Culture

The human fibrosarcoma cell line HT-1080 (CCL 121) was purchased from Sigma Aldrich (ECACC collection, Saint Quentin Fallavier, France). The human breast adenocarcinoma MCF-7 (HTB-22) and MDA-MB-231(HTB-26) and lung carcinoma A-549 (CCL-185) cell lines were purchased from the American Type Culture Collection (ATCC). Cells were cultured in MEM (HT-1080) and DMEM (A-549, MCF-7 and MDA-MB-231) with Earle salts and Glutamax I (Invitrogen, Cergy-Pontoise, France) supplemented with 10% fetal bovine serum (Invitrogen) and 1% penicillin-streptomycin (Invitrogen). Cultures were maintained at 37 °C in a humidified atmosphere containing 5% CO_2_ (*v*/*v*). Cells were routinely passaged at preconfluency using 0.05% trypsin, 0.53 mM EDTA (Invitrogen) and screened for the absence of mycoplasma using PCR method.

### 4.4. Cytotoxicity Assay

The inhibitory effects of the synthesized compounds on cell growth were assessedusing the CellTiter 96^®^ cell proliferation assay (MTS) (Promega, Charbonnieres les Bain, France). All the compounds obtained were solubilized in sterile Dimethylsulfoxide (DMSO) and then diluted to the thousandth in the culture medium. Briefly, the cells were plated at a density of 2500 cells/well in 100 µL culture medium using 96-well plates and treated with the compounds at different concentrations (6.25, 12.5, 25, 50 and 100 µM). After 24 h, 15 µL of MTS dye solution was added in each well. The plates were further incubated for 4 h. Then, 100 µL of the solubilization/stop solution was added into each well and the plate was incubated 1h at room temperature. The optical density of each well was measured at 570 nm using the microplatereader revelation 96-well multiscanner (Dynex Technologies, Chantilly, VA, USA). Data are represented as a percentage of cell growth relative to untreated cells. The IC_50_ was defined as the drug concentration required for inhibition of cell growth by 50%.

### 4.5. General Procedure for the Preparation of (R)-Carvon-Alkyne **7**

To the solution of (R)-carvoneoxime **6** (9.43 mmol) in dry acetone, potassium carbonate (28.29 mmol) and 3-bromoprop-1-yne (10.3 mmol) wereadded at 25 °C. The reaction mixture was stirred at room temperature. After 4–5 h, the solvent was evaporated under reduced pressure; the resulting residue was diluted with water and extracted withEtOAc (3 × 50 mL). The combined organic layers were dried over anhydrous Na_2_SO_4_. After evaporation of the solvent, the crude product was purified by silica gel column chromatography to give the corresponding alkyne **7**.

2-Methyl-5-(prop-1-en-2-yl)cyclohex-2-en-1-one O-prop-2-yn-1-yl oxime (**7**). Solid; yield—79%; mp—95–97 °C.

^1^H NMR (500 MHz, CDCl_3_) δ: 1.76 (3H, s, CH_3_); 1.77 (3H, s, CH_3_); 1.80–2.25 and 3.00–3.25 (5H, m, CH, CH_2_ andCH_2_); 2.37 (1H, t, *J* ≈ 5 Hz, CH); 4.61 (2H, s, CH_2_); 4.73 (2H, 2s, H_2_C=); 5.93 (1H, m, HC=). ^13^C NMR (150 MHz, CDCl_3_)δ:17.77 (CH_3_); 20.64 (CH_3_); 27.50 and 30.09 (CH_2_ and CH_2_); 40.25 (CH); 62.93 (CH_2_); 74.37 (CH); 80.20 (C); 110.03 (H_2_C=); 130.95 ©; 132.50 (HC=); 148.78 (C); 157.91 (C=N). HRMS (TOF-MS ES+) (*m*/*z*) [M+H]^+^ calculated for C_13_H_17_NO: 204.1388; found: 204.1388.

### 4.6. General Procedure for the Preparation of Carvone-1,2,3-Triazole **9a**–**h**

To a stirred solution of compound **7** (0.4 mmol) in EtOH:H_2_O (1:5, *v*/*v*) (5 mL), sodium ascorbate 0.08 mmol (20%), CuSO_4_.5H_2_O 0.06 mmol (15%) and the arylazide (**8a**–**h**) (1.2 eq, 0.48 mmol) were added in succession and stirred at room temperature for 4–5 h. After completion of the reaction (the progress of the reaction was monitored using TLC), ice cold water (100 mL) was added to the reaction mixture and extracted with EtOAc (3 × 50 mL). The combined organic layers were dried over anhydrous Na_2_SO_4_ and concentrated under reduced pressure. The obtained crude product was purified by silica gel chromatography using Hexane: EtOAc (7:3) to afford the corresponding triazole (**9a**–**h**).

(E)-2-methyl-5-(prop-1-en-2-yl)cyclohex-2-en-1-oneO-((1-phenyl-1H-1,2,3-triazol-4-yl)methyl) oxime(**9a**). Solid; yield—91%; mp127–129 °C.

^1^H NMR (500 MHz, CDCl_3_) δ (ppm): 1.73 (3H, s, CH_3_); 1.85 (3H, s, CH_3_); 1.90–2.40 and 3.10–3.20 (5H, m, CH, CH_2_ and CH_2_); 4.75 and 4.76 (2H, 2s, H_2_C=), 5.32 (2H, s, O-CH_2_); 6.02 (1H, m, HC=), 7.40–7.80 (5H, m, HC_Ar_); 8.00 (1H, s, C_5′_H).^13^C NMR (150 MHz, CDCl_3_) δ (ppm): 17.72 (CH_3_); 20.65 (CH_3_); 27.60 and 30.80 (CH_2_ and CH_2_); 40.33 (CH); 67.58 (O-CH_2_); 110.17 (H_2_C=); 120.64 (HC_Ar_); 121.74 (CH_5′_); 128.71 (HC_Ar_); 129.72 (HC_Ar_); 130.35 (C); 132.83 (HC=); 137.59 (C_Ar_); 145.77 (C) 147.88 (C); 156.93 (C=N). HRMS (TOF-MS ES+) (*m*/*z*) [M+H]^+^ calculated forC_19_H_22_N_4_O: 323.1872; found: 323.1873.

(E)-2-methyl-5-(prop-1-en-2-yl)cyclohex-2-en-1-oneO-((1-(p-tolyl)-1H-1,2,3-triazol-4-yl)methyl) oxime(**9b**). Solid; yield—87%; mp—145–146 °C.

^1^H NMR (500 MHz, CDCl_3_) δ (ppm): 1.66 (3H, s, CH_3_); 1.78 (3H, s, CH_3_); 1.90–2.30 and 3.10–3.20 (5H, m, CH, CH_2_ and CH_2_); 2.35 (3H, s, CH_3_); 4.67 and 4.68 (2H, 2s, H_2_C=), 5.28 (2H, s, O-CH_2_); 5.96 (1H, m, HC=), 7.20–7.60 (4H, m, HC_Ar_); 7.90 (1H, s, C_5′_H).^13^C NMR (150 MHz, CDCl_3_) δ (ppm): 17.70 (CH_3_); 20.64 (CH_3_); 21.10 (CH_3_); 27.99 and 30.32 (CH_2_ and CH_2_); 40.33 (CH); 67.27 (O-CH_2_); 109.94 (H_2_C=); 120.55 (HC_Ar_); 121.32 (CH_5′_); 130.20 (HC_Ar_); 132.76 (HC=); 130.38 (C); 138.80 (C_Ar_); 135.30 (C_Ar_); 145.58 (C) 146.88 (C); 156.88 (C=N). HRMS (TOF-MS ES+) (*m*/*z*) [M+H]^+^ calculated for C_20_H_24_N_4_O: 337.2028; found: 337.2031.

(E)-2-methyl-5-(prop-1-en-2-yl)cyclohex-2-en-1-oneO-((1-(4-chlorophenyl)-1H-1,2,3-triazol-4-yl)methyl) oxime(**9c**). Solid; yield—92%; mp—109–111 °C.

^1^H NMR (500 MHz, CDCl_3_) δ (ppm): 1.72 (3H, s, CH_3_); 1.84 (3H, s, CH_3_); 1.90–2.40 and 3.05–3.20 (5H, m, CH, CH_2_ and CH_2_); 4.73 and 4.76 (2H, 2s, H_2_C=), 5.31 (2H, s, O-CH_2_); 6.01 (1H, m, HC=), 7.40–7.80 (4H, m, HC_Ar_); 7.90 (1H, s, C_5′_H).^13^C NMR (150 MHz, CDCl_3_) δ (ppm): 17.73 (CH_3_); 20.65 (CH_3_); 27.50 and 30,91 (CH_2_ and CH_2_); 40.31 (CH); 66.98 (O-CH_2_); 110.37 (H_2_C=); 121.20 (CH_5′_); 121.76 (HC_Ar_); 129.91 (HC_Ar_); 130.29 (C_Ar_); 132.94 (HC=); 134.48 (C_Ar_); 131.53 (C); 146.28 (C) 147.85 (C); 157.01 (C=N). HRMS (TOF-MS ES+) (*m*/*z*) [M + H]^+^ calculated for C_19_H_21_ClN_4_O: 357.1482; found: 357.1482.

(E)-2-methyl-5-(prop-1-en-2-yl)cyclohex-2-en-1-one O-((1-(4-nitrophenyl)-1H-1,2,3-triazol-4-yl)methyl) oxime(**9d**). Solid; yield—88%; mp—131–133 °C.

^1^H NMR (500 MHz, CDCl_3_) δ (ppm): 1.52 (3H, s, CH_3_); 1.66 (3H, s, CH_3_); 1.90–2.30 and 3.00–3.20 (5H, m, CH, CH_2_ and CH_2_); 4.67 and 4,70 (2H, 2s, H_2_C=), 5.26 (2H, s, O-CH_2_); 5.96 (1H, m, HC=); 7.80–8.10 (4H, m, HC_Ar_); 8.35 (1H, s, C_5′_H).^13^C NMR (150 MHz, CDCl_3_) δ (ppm): 17.74 (CH_3_); 20.66 (CH_3_); 28.00 and 30.31 (CH_2_ and CH_2_); 40.30 (CH); 66.97 (O-CH_2_); 109.99 (H_2_C=); 120.51 (HC_Ar_); 121.05 (CH_5′_); 125.51 (HC_Ar_); 130.21 (C); 133.14 (HC=); 141.24 (C_Ar_); 146.83 (C_Ar_); 147.20 (C) 147.80 (C); 157.21 (C=N). HRMS (TOF-MS ES+) (*m*/*z*) [M + H]^+^ calculated for C_19_H_21_N_5_O_3_: 368.1723; found: 368.1712.

(E)-2-methyl-5-(prop-1-en-2-yl)cyclohex-2-en-1-oneO-((1-(o-tolyl)-1H-1,2,3-triazol-4-yl)methyl) oxime(**9e**). Solid; yield—82%; mp—120–122 °C.

^1^H NMR (500 MHz, CDCl_3_) δ (ppm): 1.66 (3H, s, CH_3_); 1.77 (3H, s, CH_3_); 1.90–2.30 and 3.00–3.20 (5H, m, CH, CH_2_and CH_2_); 2.15 (3H, s, CH_3_); 4.67 and 4.69 (2H, 2s, H_2_C=), 5.26 (2H, s, O-CH_2_); 5.95 (1H, m, HC=), 7.00–7.50 (4H, m, HC_Ar_); 7.70 (1H, s, C_5′_H).^13^C NMR (150 MHz, CDCl_3_) δ (ppm): 17.66 (CH_3_); 20.65 (CH_3_); 17.88 (CH_3_); 27.99 and 30.32 (CH_2_ and CH_2_); 40.34 (CH); 67.29 (O-CH_2_); 109.96 (H_2_C=); 124,66 (CH_5′_); 126.04 (HC_Ar_); 126.81 (HC_Ar_); 129.79 (HC_Ar_); 131.45 (HC_Ar_); 130.80 (C_Ar_); 132.75 (HC=); 133.69 (C); 136.59 (C_Ar_); 144.93 (C); 147.89 (C); 156.89 (C=N). HRMS (TOF-MS ES+) (*m*/*z*) [M + H]^+^ calculated for C_20_H_24_N_4_O: 337.2028; found: 337.2022.

(E)-2-methyl-5-(prop-1-en-2-yl)cyclohex-2-en-1-oneO-((1-(4-chloro-2-methylphenyl)-1H-1,2,3-triazol-4-yl)methyl) oxime(**9f**). Solid; yield—86%; mp115–117 °C.

^1^H NMR (500 MHz, CDCl_3_) δ (ppm): 1.66 (3H, s, CH_3_); 1.75 (3H, s, CH_3_); 2.13 (3H, s, CH_3_); 1.90–2.10 and 2.16–3.20 (5H, m, CH, CH_2_ and CH_2_); 4.67 and 4.69 (2H, 2s, H_2_C=), 5.26 (2H, s, O-CH_2_); 5.94 (1H, m, HC=), 7.10–7.60 (3H, m, HC_Ar_); 7.68 (1H, s, C_5′_H). ^13^C NMR (150 MHz, CDCl_3_) δ (ppm): 15.38 (CH_3_); 17.66 (CH_3_); 20.65 (CH_3_); 27.98 and 30.31 (CH_2_ and CH_2_); 40.32 (CH); 67.18 (O-CH_2_); 109.97 (H_2_C=); 124.89 (CH_5′_); 124.89 (HC_Ar_); 127.12 (C_Ar_); 130.80 (C); 130.80 (HC=); 132.86 (HC_Ar_); 136.04 (C_Ar_); 137.69 (C_Ar_); 145.21 (C) 147.86 (C); 156.98 (C=N). HRMS (TOF-MS ES+) (*m*/*z*) [M + H]^+^ calculated for C_20_H_23_ClN_4_O: 371.1620; found: 371.1629.

(E)-2-methyl-5-(prop-1-en-2-yl)cyclohex-2-en-1-oneO-((1-(4-fluorophenyl)-1H-1,2,3-triazol-4-yl)methyl) oxime(**9g**). Solid; yield—94%; mp—130–132 °C.

^1^H NMR (500 MHz, CDCl_3_) δ (ppm): 1.72 (3H, s, CH_3_); 1.85 (3H, s, CH_3_); 1.90–2.40 and 3.10–3.20 (5H, m, CH, CH_2_ and CH_2_); 4.73 and 4.76 (2H, 2s, H_2_C=), 5.31 (2H, s, O-CH_2_); 6.08 (1H, m, HC=), 7.10–7.80 (4H, m, HC_Ar_); 7.95 (1H, s, C_5′_H).^13^C NMR (150 MHz, CDCl_3_) δ (ppm): 17.71 (CH_3_); 20.64 (CH_3_); 27.50 and 30.09 (CH_2_ and CH_2_); 40.32 (CH); 67.28 (O-CH_2_); 109.96 (H_2_C=); 116.53 (HC_Ar_); 121.46 (CH_5′_); 122.54 (HC_Ar_); 130.31 (C); 132.89 (HC=); 136.73 (C_Ar_); 133.39 (C_Ar_); 145.93 (C) 147.86 (C); 156.97 (C=N). HRMS (TOF-MS ES+) (*m*/*z*) [M + H]^+^ calculated for C_19_H_21_FN_4_O: 341.1778; found: 341.1783.

(E)-2-methyl-5-(prop-1-en-2-yl)cyclohex-2-en-1-oneO-((1-benzyl-1H-1,2,3-triazol-4-yl)methyl) oxime(**9h**). Solid; yield—89%; mp—105–107 °C.

^1^H NMR (500 MHz, CDCl_3_) δ (ppm): 1.72 (3H, s, CH_3_); 1.79 (3H, s, CH_3_); 1.90–2.40 and 3.00–3.25 (5H, m, CH, CH_2_ and CH_2_); 4.73 and 4.76 (2H, 2s, H_2_C=), 5.22 (2H, s, CH_2_); 5.54 (2H, s, O-CH_2_); 5.99 (1H, m, HC=), 7.25–7.40 (5H, m, HC_Ar_); 7.50 (1H, s, C_5′_H). ^13^C NMR (150 MHz, CDCl_3_) δ (ppm): 17.60 (CH_3_); 20.63 (CH_3_); 27.94 and 30.29 (CH_2_ and CH_2_); 40.33 (CH); 54.09 (CH_2_); 67.25 (O-CH_2_); 109.91 (H_2_C=); 123.10 (CH_5′_); 128.05 (HC_Ar_); 128.70 (HC_Ar_); 129.08 (HC_Ar_); 130.38 (C_Ar_); 132.62 (HC=); 134.69 (C); 145.90 (C) 147.90 (C); 156.74 (C=N). HRMS (TOF-MS ES+) (*m*/*z*) [M + H]^+^ calculated for C_20_H_24_N_4_O: 337.2028; found: 337.2037.

## Figures and Tables

**Table 1:** Isolated yields and mass spectroscopic data of the terpenic 1,2,3-triazoles **9a**–**h**.

Product	Formula	Ar	Yield (%)	[MH]^+ (a)^	[MH]^+ (b)^
**9a**	C_19_H_22_N_4_O	C_6_H_5_	91	323.1872	323.1873
**9b**	C_20_H_24_N_4_O	4-CH_3_-C_6_H_4_	87	337.2028	337.2031
**9c**	C_19_H_21_ClN_4_O	4-Cl-C_6_H_4_	92	357.1482	357.1482
**9d**	C_19_H_21_N_5_O_3_	4-NO_2_-C_6_H_4_	88	368.1723	368.1712
**9e**	C_20_H_24_N_4_O	2-CH_3_-C_6_H_4_	82	337.2028	337.2022
**9f**	C_20_H_23_ClN_4_O	2-CH_3_-4-Cl-C_6_H_3_	86	371.1620	371.1629
**9g**	C_19_H_21_FN_4_O	4-F-C_6_H_4_	94	341.1778	341.1783
**9h**	C_20_H_24_N_4_O	C_6_H_5_-CH_2_	89	337.2028	337.2037

^(a)^ Calculated mass; ^(b)^ found mass.

**Table 2:** NMR characteristic signals of terpenic 1,2,3-triazoles **9a**–**h**.

Compound	9a	9b	9c	9d	9e	9f	9g	9h
**H-C3= (δ ^1^H)**	6.02	5.96	6.01	5.96	5.95	5.94	6.08	5.99
**=C3 (δ ^13^C)**	132.83	132.76	132.94	133.14	132.75	130.80	132.89	132.62
**=CH_2_ (δ^1^H)**	4.75	4.67	4.73	4,70	4.69	4.69	4.76	4.76
**=CH_2_ (δ^13^C)**	110.17	109.94	110.37	109.99	109.96	109.97	109.96	109.91
**H-C5′ (δ^1^H)**	8.00	7.90	7.90	8.35	7.70	7.68	7.95	7.50
**C5′ (δ^13^C)**	121.74	121.32	121.20	121.05	124,66	124.89	121.46	123.10
**C4′ (δ^13^C)**	145.77	145.58	146.28	147.20	144.93	145.21	145.93	145.90

**Table 3:** Electronic chemical potential µ(eV), chemical hardness η(eV), global electrophilicity ω(eV), and the nucleophilicity N(eV) of phenylazide (**8a**), acetylene carvone derivative (**7**) and dinucear Cu(I)-acetylide (**2Cu(I)-7**).

	µ	η	ω	N
**8a**	−3.62	5.16	1.27	2.92
**7**	−3.34	5.22	1.07	3.17
**2Cu(I)-7**	−6.21	4.36	4.42	0.73

**Table 4:** Relative energies (∆E, kcal/mol), enthalpies (∆H, kcal/mol), entropies (∆S, kcal/mol, at 25°C) and Gibbs energies (∆G, kcal/mol) for all the stationary points relative to the reactants.

Species	∆E	∆H	T∆S	∆G
**8a + 2Cu(I)-7**	0	0	0	0
**1,4-RC**	−15.39	−14.79	−0.0175	−14.77
**1,4-TS1**	−9.92	−10.34	−0.0231	−10.32
**1,4-In**	−25.11	−25.67	−0.0238	−25.65
**1,4-TS2**	−11.68	−12.34	−0.1013	−12.24
**1,4-P**	−57.29	−57.71	−0.1150	−57.59
**1,5-RC**	−17.65	−16.75	−0.1006	−16.65
**1,5-TS1**	12.46	12.71	−0.1067	12.82
**1,5-In**	−21.48	−21.75	−0.0009	−21.75
**1,5-TS2**	−18.79	−19.45	−0.0094	−19.44
**1,5-P**	−52.37	−52.78	−0.1249	−52.65

**Table 5:** In vitro antiproliferative activity of the compounds **7** and **9a**–**h**.

Compound	IC_50_ (µM)			
	HT1080	A-549	MCF-7	MDA-MB-231
**7**	>100	>100	>100	>100
**9a**	85.76	71.21	55.45	72.63
**9b**	51.9	>100	89.75	>100
**9c**	45.19	>100	25.03	37.5
**9d**	25.77	30.61	27.89	45.28
**9e**	>100	>100	>100	>100
**9f**	>100	62.98	87.21	>100
**9g**	30.44	51.83	30.11	>100
**9h**	48.69	>100	41.54	39.45
**Dox**	5.09	6.41	5.41	5.1

## Data Availability

Available from authors.

## Supplementary Materials

The following are available online, Supporting experimental information, Table S1: The calculated energies (E. a.u). zero-point vibrational energies (ZPE. a.u). thermal corrections (TCE. a.u). entropy values (S. cal/mol/K). ZPE corrected energies (Ecorr. a.u). enthalpies (H. a.u). and T S (a.u). at 25 °C. for all possible regioisomers of the 32CA between 7 and 8a, Table S2: Energies (E). enthalpies (H) and Gibbs free energies (G) for stationary points of all possible regioisomers of the 32CA between 7 and 8a relative to the separate reactants. All values are in kcal/mol, Table S3: The calculated energies (E. a.u), zero-point vibrational energies (ZPE. a.u), thermal corrections (TCE. a.u), entropy values (S. cal/mol/K), ZPE corrected energies (Ecorr. a.u), enthalpies (H. a.u) and T S (a.u). at 25 °C, for the stationary points of the two regioisomers 1,4- and 1,5-disubstited triazole of the 32CA between 2Cu(I)-7 and 8a. Table S4: Table S4. Energies (E. a.u), zero-point vibrational energies (ZPE. a.u), thermal corrections (TCE. a.u), entropy values (S. cal/mol/K), ZPE corrected energies (Ecorr. a.u), enthalpies (H. a.u) and T S (a.u) at 25 °C calculated at B3LYP/6-31G* in ethanol as solvent using the PCM model for the stationary points of the two regioisomers 1,4 and 1,5-disubstituted 1,2,3-triazole of the 32CA between 2Cu(I)-7 and 8a.

## Sample Availability

Samples of the compounds are available from the authors.
