# Supplementary material for: New 1,2,3-Triazoles from (R)-Carvone: Synthesis, DFT Mechanistic Study and In Vitro Cytotoxic Evaluation
Source: Molecules. 2022 Jan 25;27(3):769. doi: 10.3390/molecules27030769 (PMC8839216; doi:10.3390/molecules27030769)
Supplement: Supplementary file 1 [file molecules-27-00769-s001.zip › molecules-1402399-supplementary.pdf]

New 1,2,3-triazoles from (R)-carvone : Synthesis, DFT mechanistic study and *in vitro* cytotoxic evaluation.

Ali Oubella[1], Abdoullah Bimoussa[1], Abdellah N'ait oussidi[1], Mourad Fawzi[1], Aziz Auhmani[1], Hamid Morjani[2], Abdelkhalek Riahi[3], M'hamed Esseffar*[1], Carol Parish[4], My Youssef AitItto*[1]

I- Experimental Supporting Information

Spectral data for product 8:

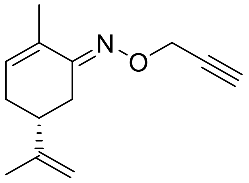

8

NMR Spectroscopy (500 MHz, $CDCl_3$)

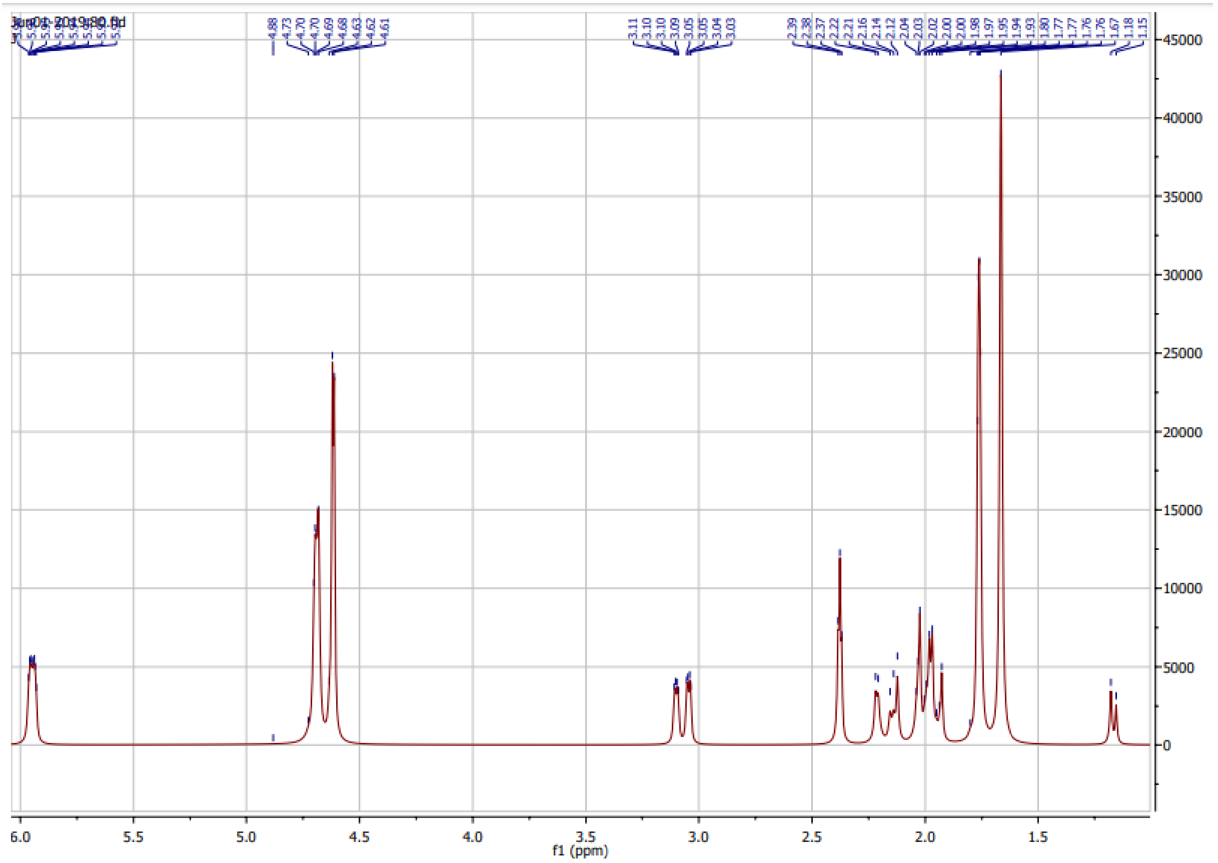

^{1}H NMR spectrum

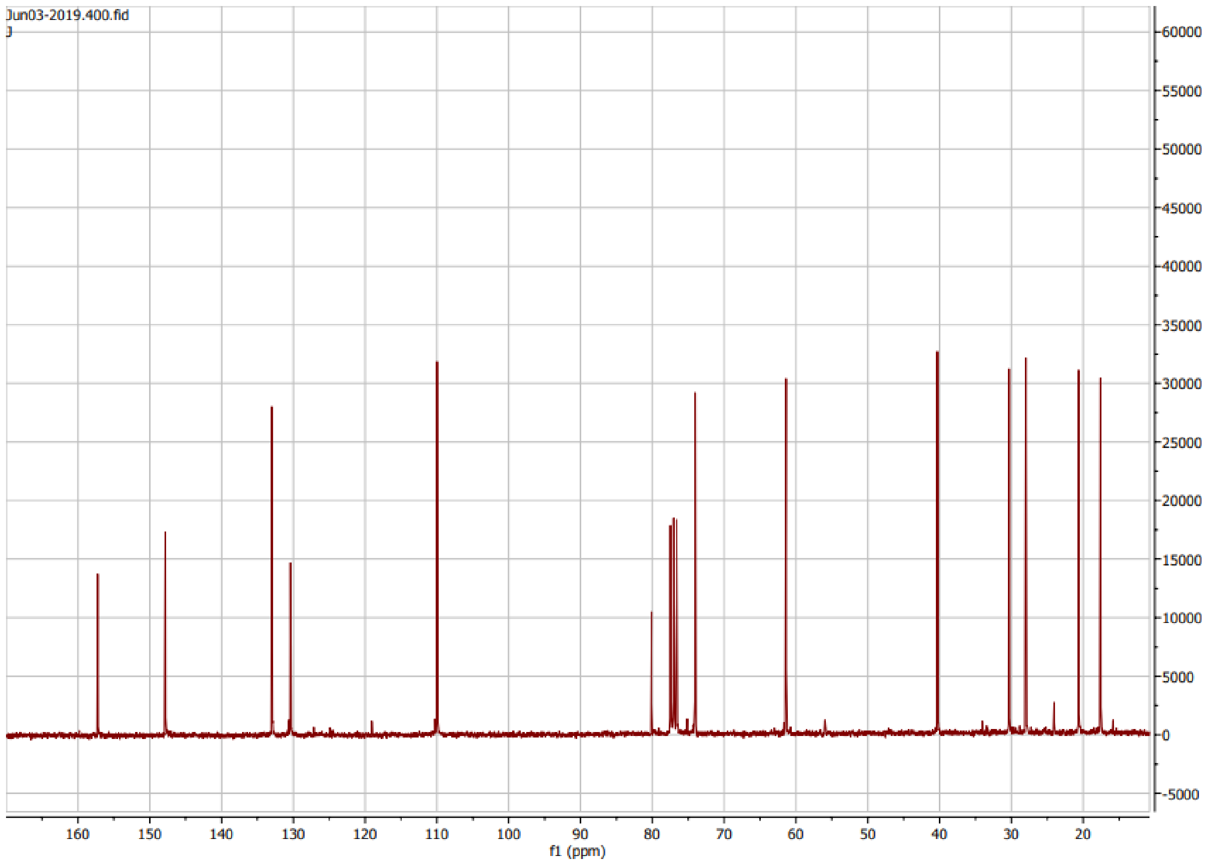

^{13}C Decoupled ^{1}H NMR spectrum

HRMS spectrum

Elemental Composition Report Page 1

Single Mass Analysis
Tolerance = 5.0 PPM / DBE: min = -1.0, max = 16.0
Element prediction: Off
Number of isotope peaks used for i-FIT = 4

Monoisotopic Mass, Even Electron Ions
89 formula(e) evaluated with 1 results within limits (all results (up to 1000) for each mass)
Elements Used:
C: 0-500 H: 0-1000 N: 0-4 O: 0-6

AB120 1:6
11-Dec-2019
1912123 546 (3.303) Cm (545:549-(495:501+628:633)) 1: TOF MS ES+ 7.00e+002

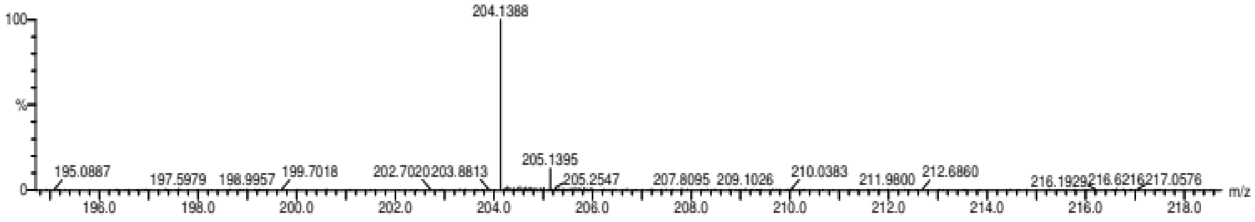

Minimum:				-1.0			
Maximum:		5.0	5.0	16.0			
Mass	Calc. Mass	mDa	PPM	DBE	i-FIT	i-FIT (Norm)	Formula
204.1388	204.1388	0.0	0.0	5.5	163.8	0.0	C13 H18 N O

Spectral data for 123-triazole-Carvone9a-h:

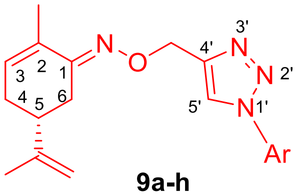

9a-h

9a : Ar= C_6H_5
9b : Ar= 4-CH_3-C_6H_4
9c : Ar= 4-Cl-C_6H_4
9d : Ar= 4-NO_2-C_6H_4
9e : Ar= 2-CH_3-C_6H_4
9f : Ar= 2-CH_3-4Cl-C_6H_3
9g : Ar= 4-F-C_6H_4
9h : Ar= C_6H_5-CH_2

1,2,3-triazole-Carvone 9a:

NMR Spectroscopy (500 MHz, $CDCl_3$)

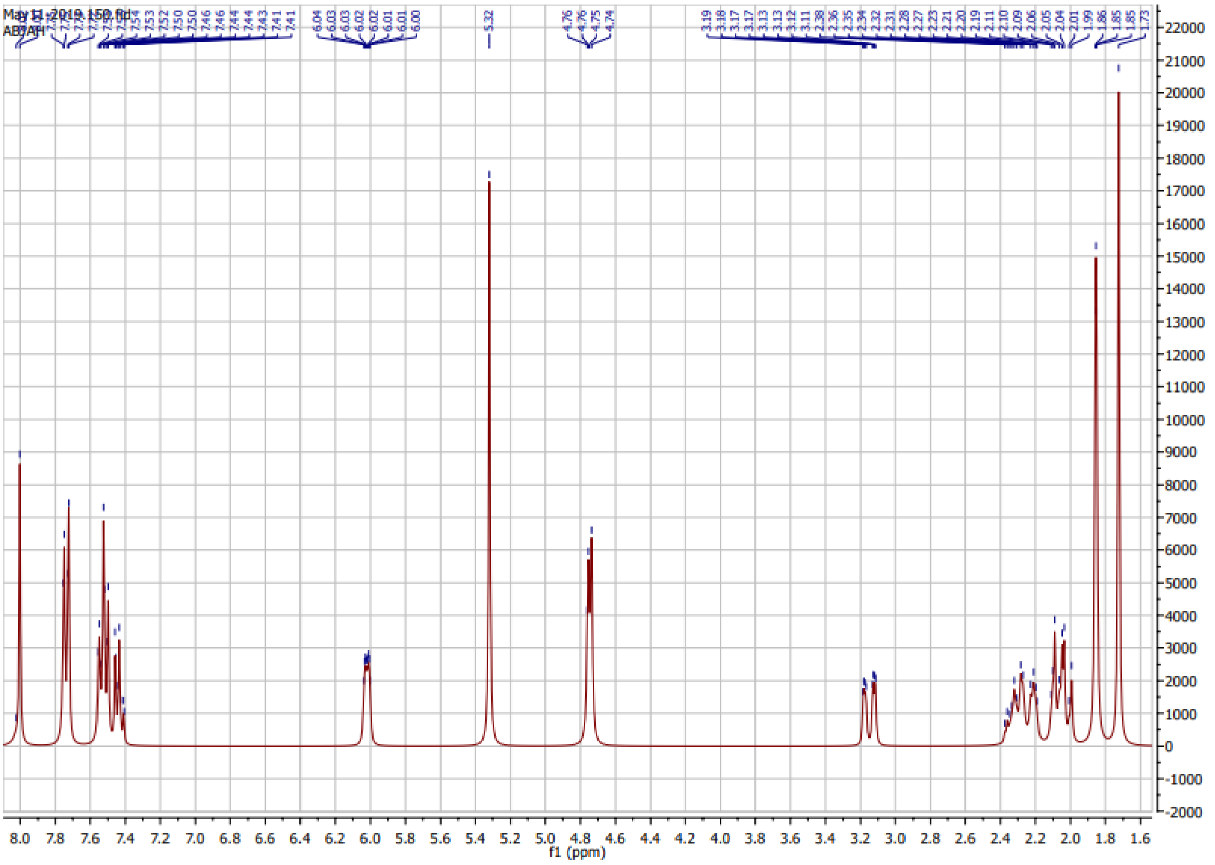

^{1}H NMR spectrum

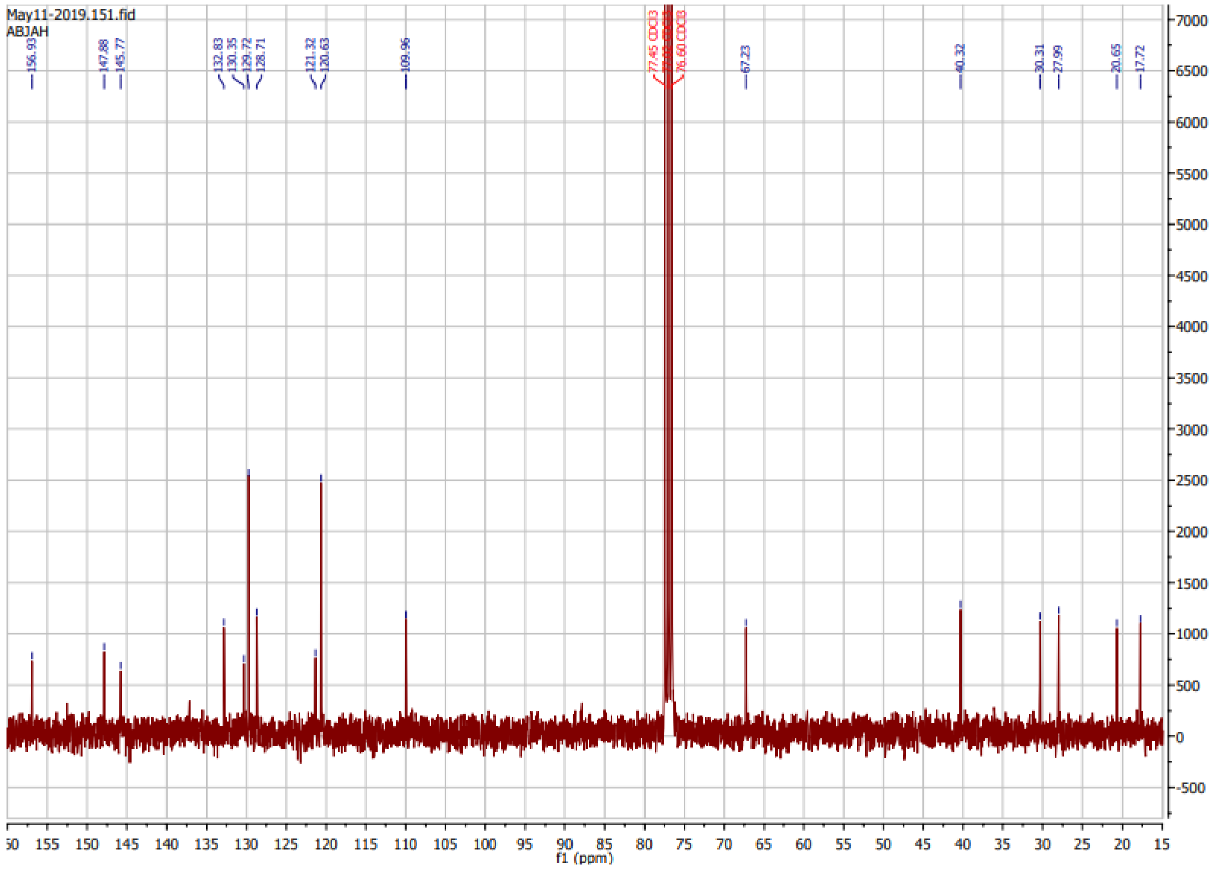

^{13}C Decoupled^{1}H NMR spectrum

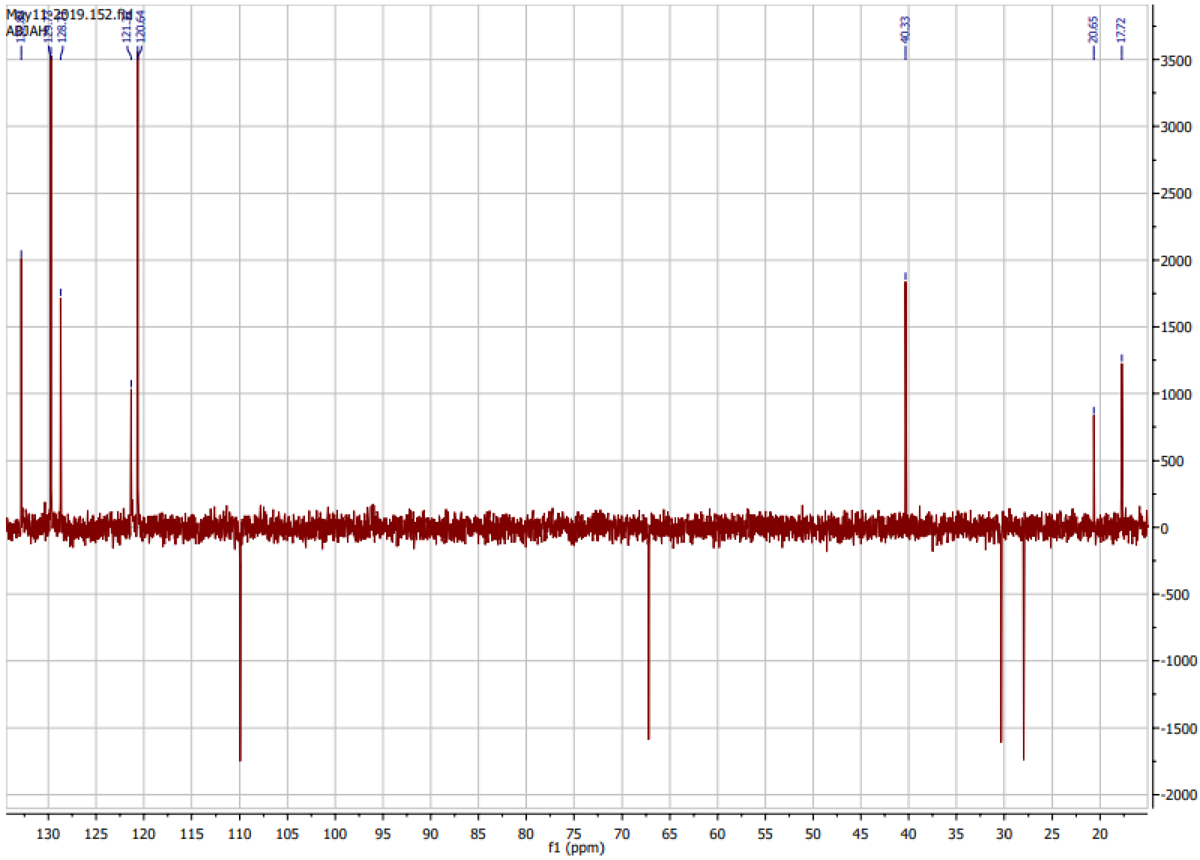

^{13}C Decoupled^{1}H NMR spectrum DEPT 135 Mode

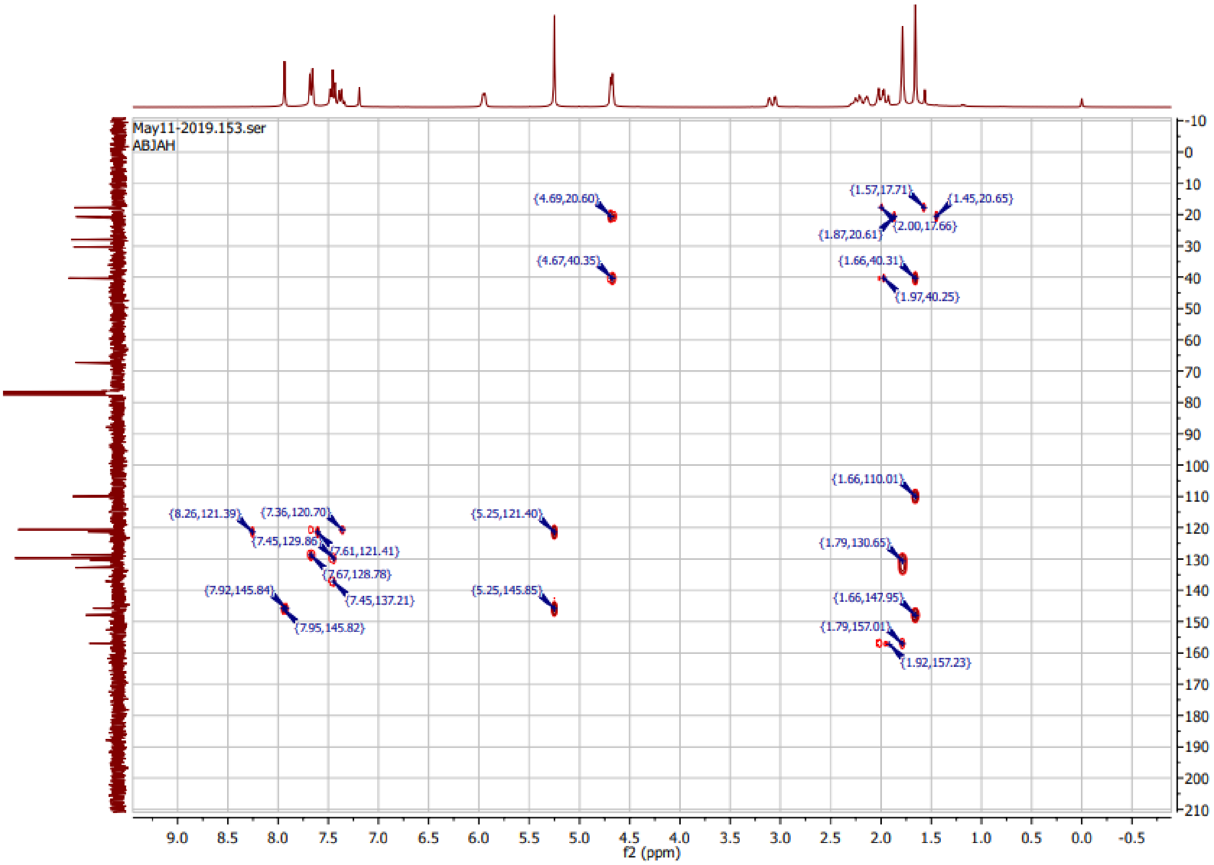

HMBC Spectrum

HRMS spectrum

Elemental Composition Report **Page 1**

Single Mass Analysis
Tolerance = 5.0 PPM / DBE: min = -1.0, max = 16.0
Element prediction: Off
Number of isotope peaks used for i-FIT = 4

Monoisotopic Mass, Even Electron Ions
143 formula(e) evaluated with 2 results within limits (all results (up to 1000) for each mass)
Elements Used:
C: 0-500 H: 0-1000 N: 0-4 O: 0-6

AB121 1:7

11-Dec-2019
1912124 571 (3.451) Cm (571:574-(513:515+647))

1: TOF MS ES+
3.91e+003

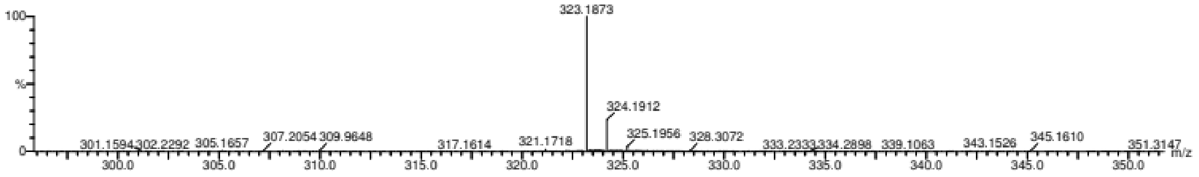

Minimum:				-1.0				
Maximum:		5.0	5.0	16.0				
Mass	Calc. Mass	mDa	PPM	DBE	i-FIT	i-FIT (Norm)	Formula	
323.1873	323.1872	0.1	0.3	10.5	338.7	0.1	C19 H23 N4 O	
	323.1858	1.5	4.6	5.5	340.8	2.2	C18 H27 O5	

1,2,3-triazole-Carvone 9b:

NMR Spectroscopy (500 MHz, $CDCl_3$)

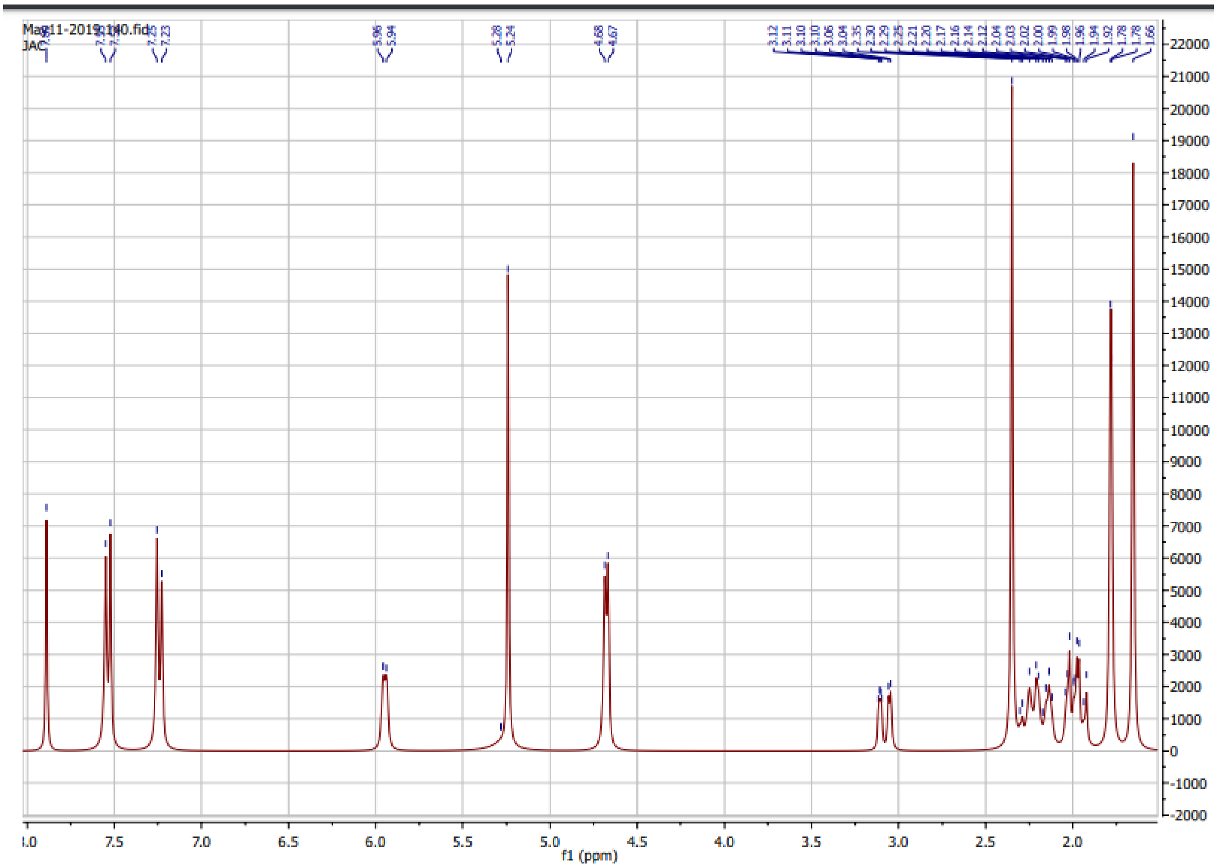

^1H NMR spectrum

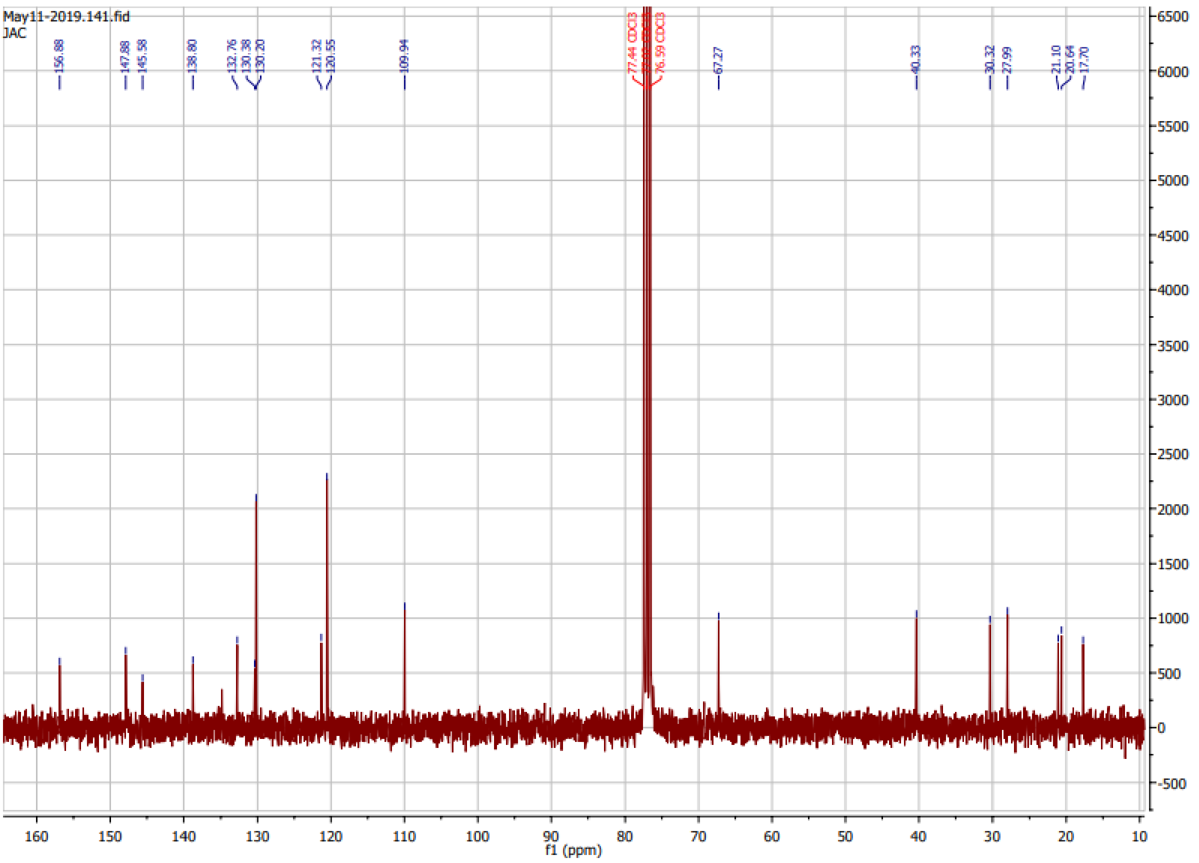

^{13}C Decoupled^{1}H NMR spectrum

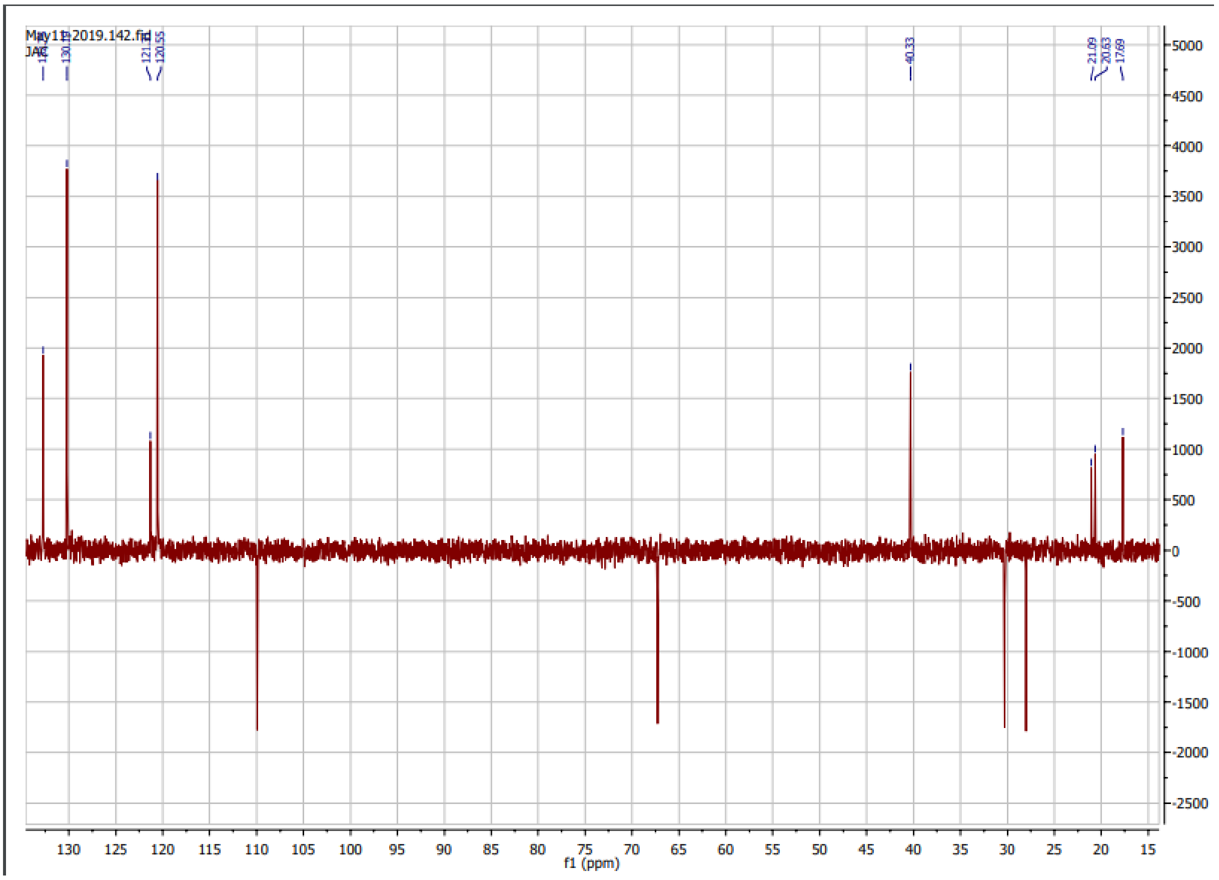

^{13}C Decoupled ^{1}H NMR spectrum DEPT 135 Mode

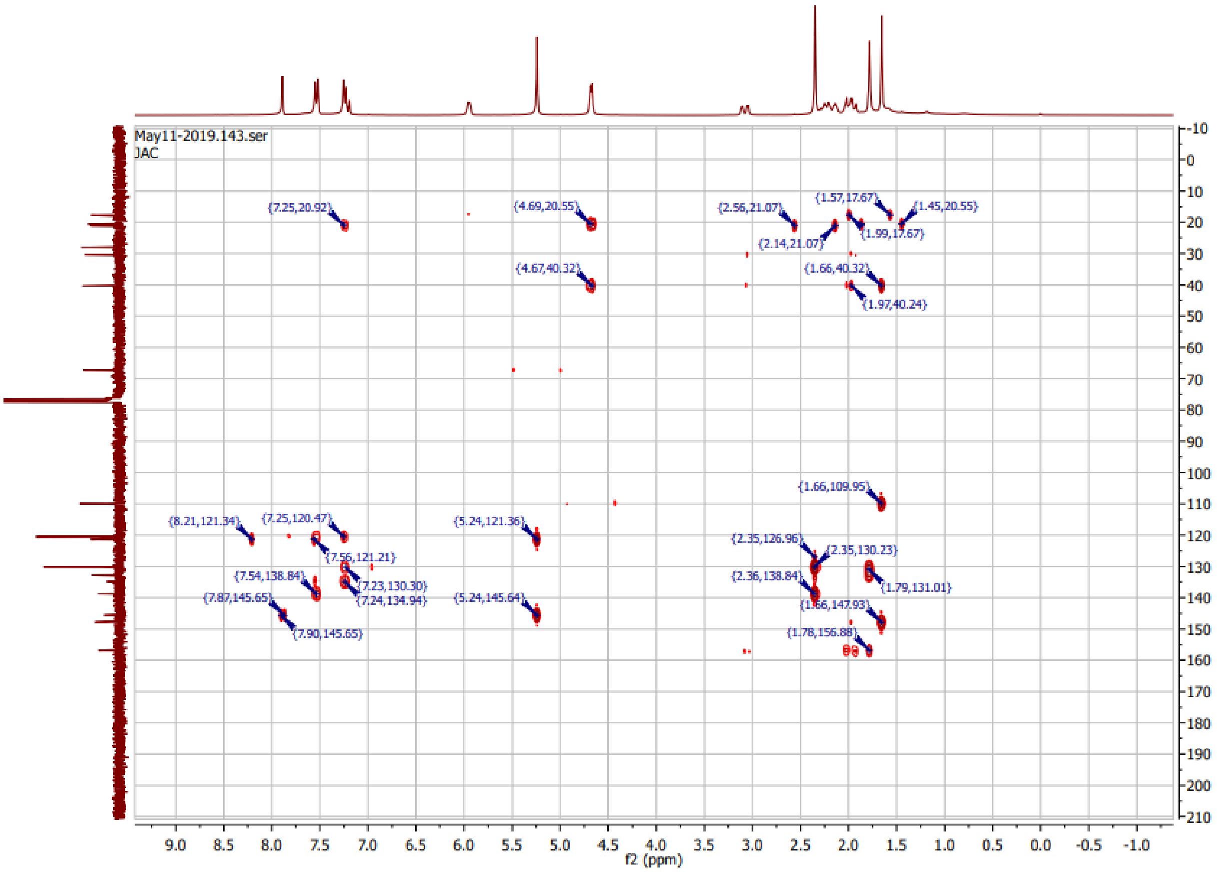

HMBC Spectrum

HRMS spectrum

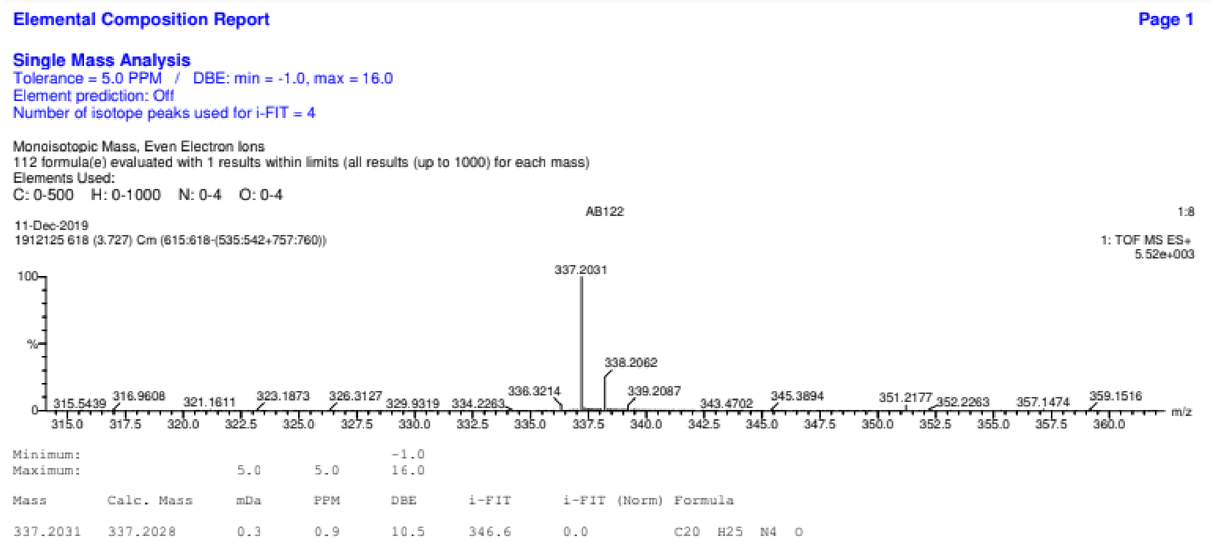

Elemental Composition Report **Page 1**

Single Mass Analysis
Tolerance = 5.0 PPM / DBE: min = -1.0, max = 16.0
Element prediction: Off
Number of isotope peaks used for i-FIT = 4

Monoisotopic Mass, Even Electron Ions
112 formula(e) evaluated with 1 results within limits (all results (up to 1000) for each mass)
Elements Used:
C: 0-500 H: 0-1000 N: 0-4 O: 0-4

AB122 1:8
11-Dec-2019
1912125 618 (3.727) Cm (615:618-(535:542+757:760)) 1: TOF MS ES+ 5.52e+003

Minimum:				-1.0			
Maximum:		5.0	5.0	16.0			
Mass	Calc. Mass	mDa	PPM	DBE	i-FIT	i-FIT (Norm)	Formula
337.2031	337.2028	0.3	0.9	10.5	346.6	0.0	C20 H25 N4 O

1,2,3-triazole-Carvone 9c:

NMR Spectroscopy (500 MHz, $CDCl_3$)

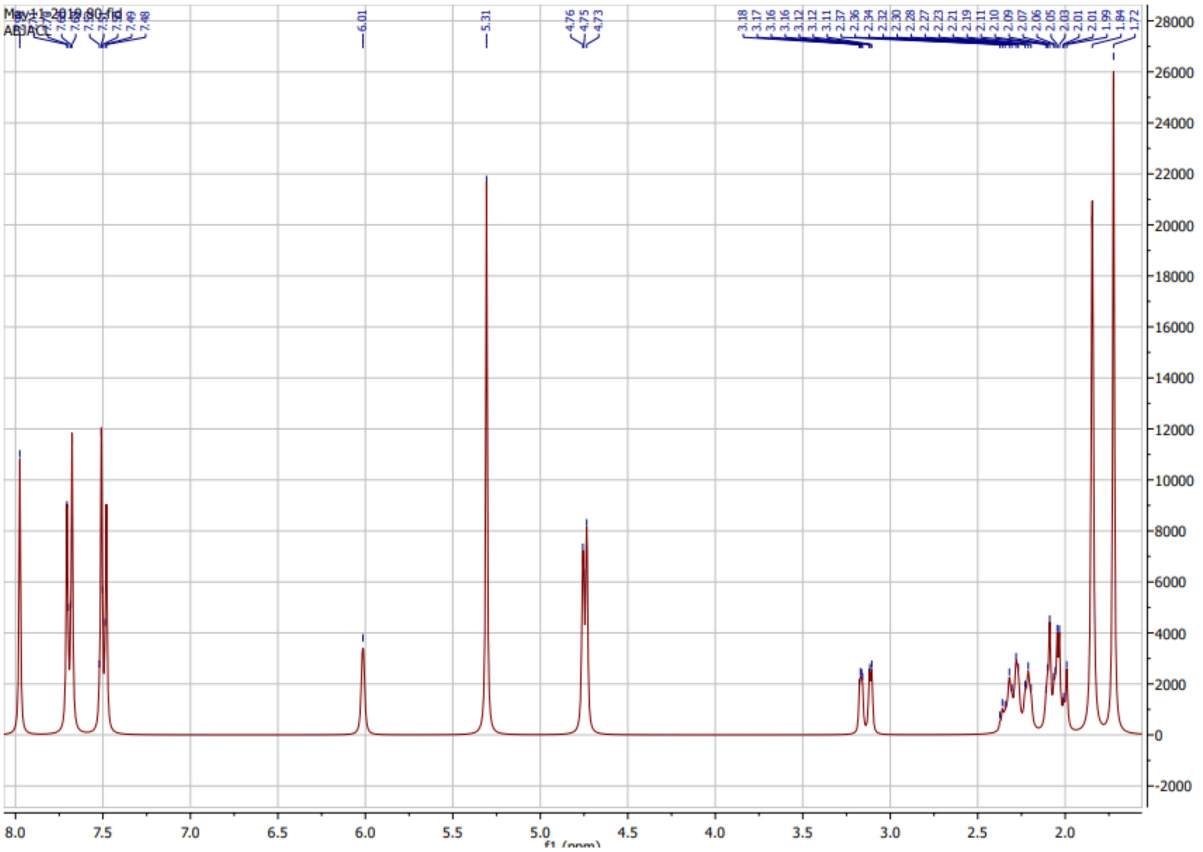

^{1}H NMR spectrum

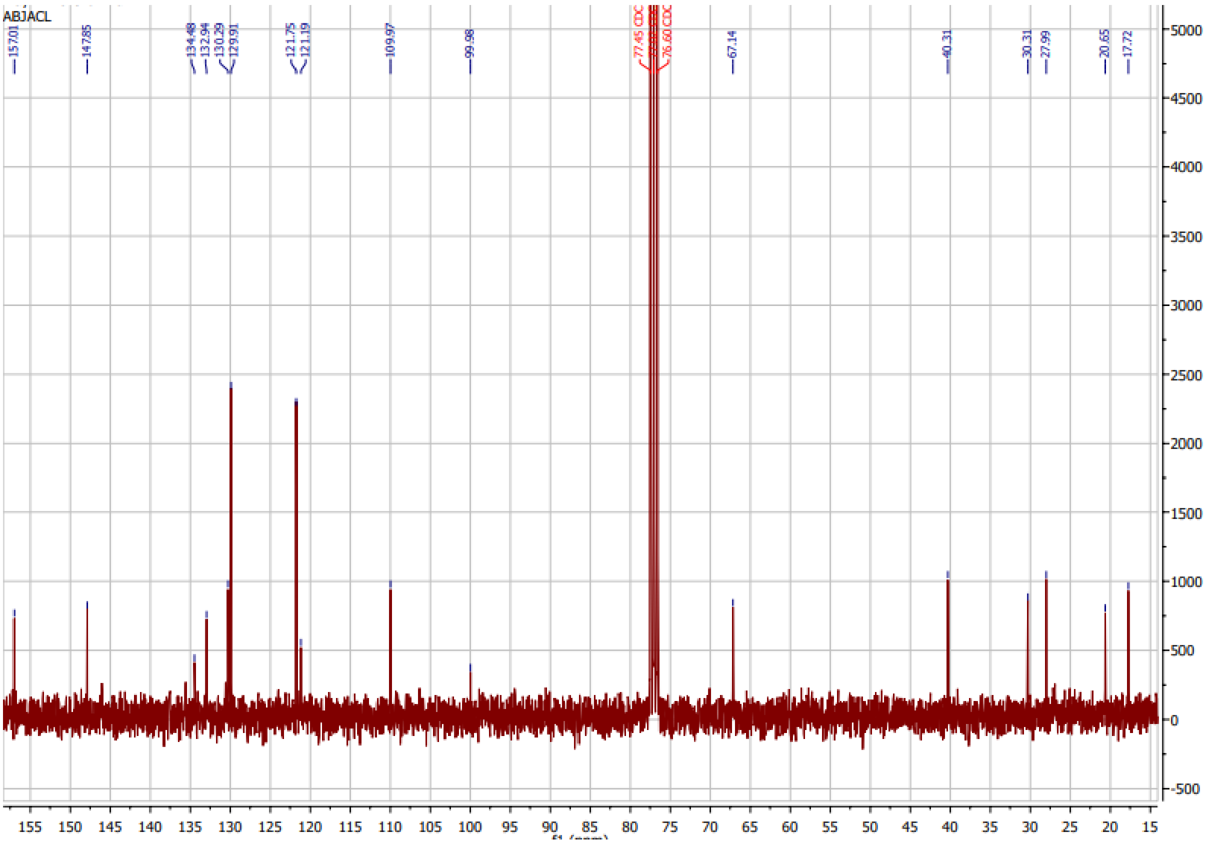

^{13}C Decoupled^{1}H NMR spectrum

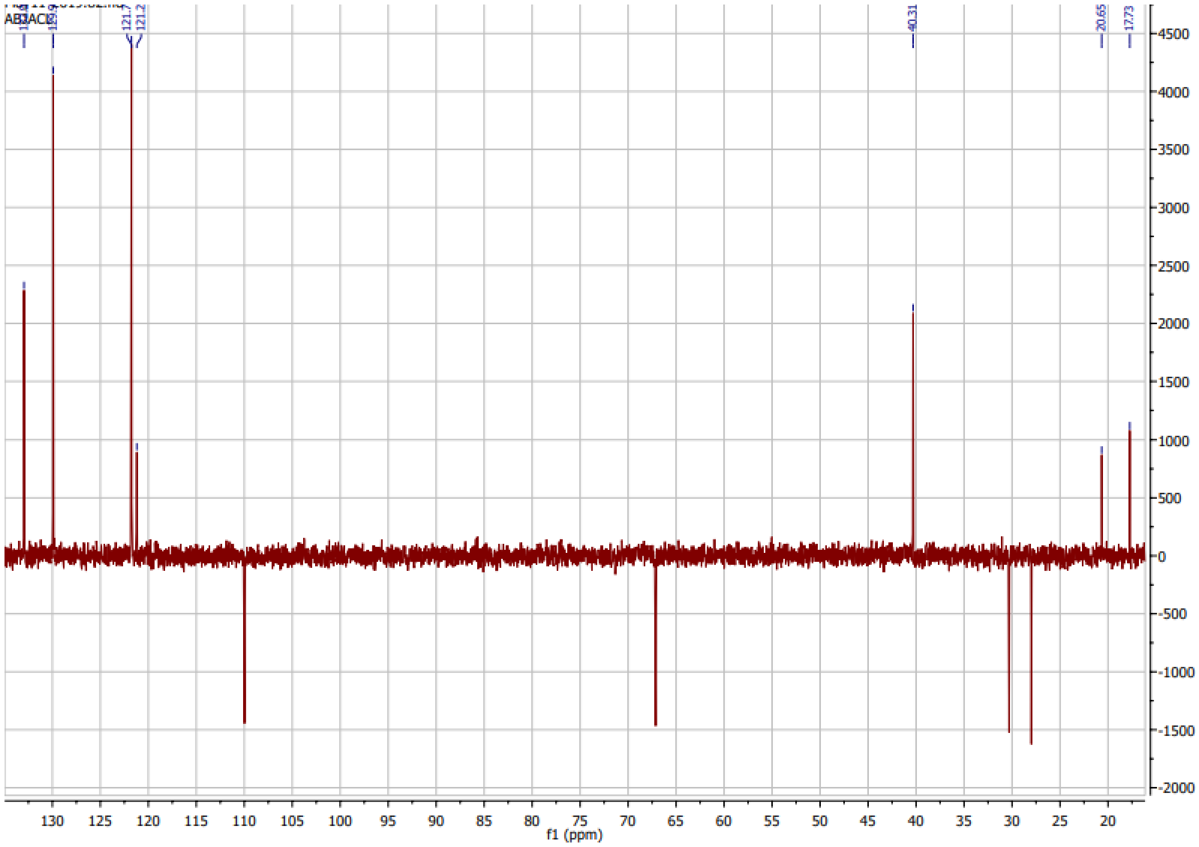

^{13}C Decoupled^{1}H NMR spectrum DEPT 135 Mode

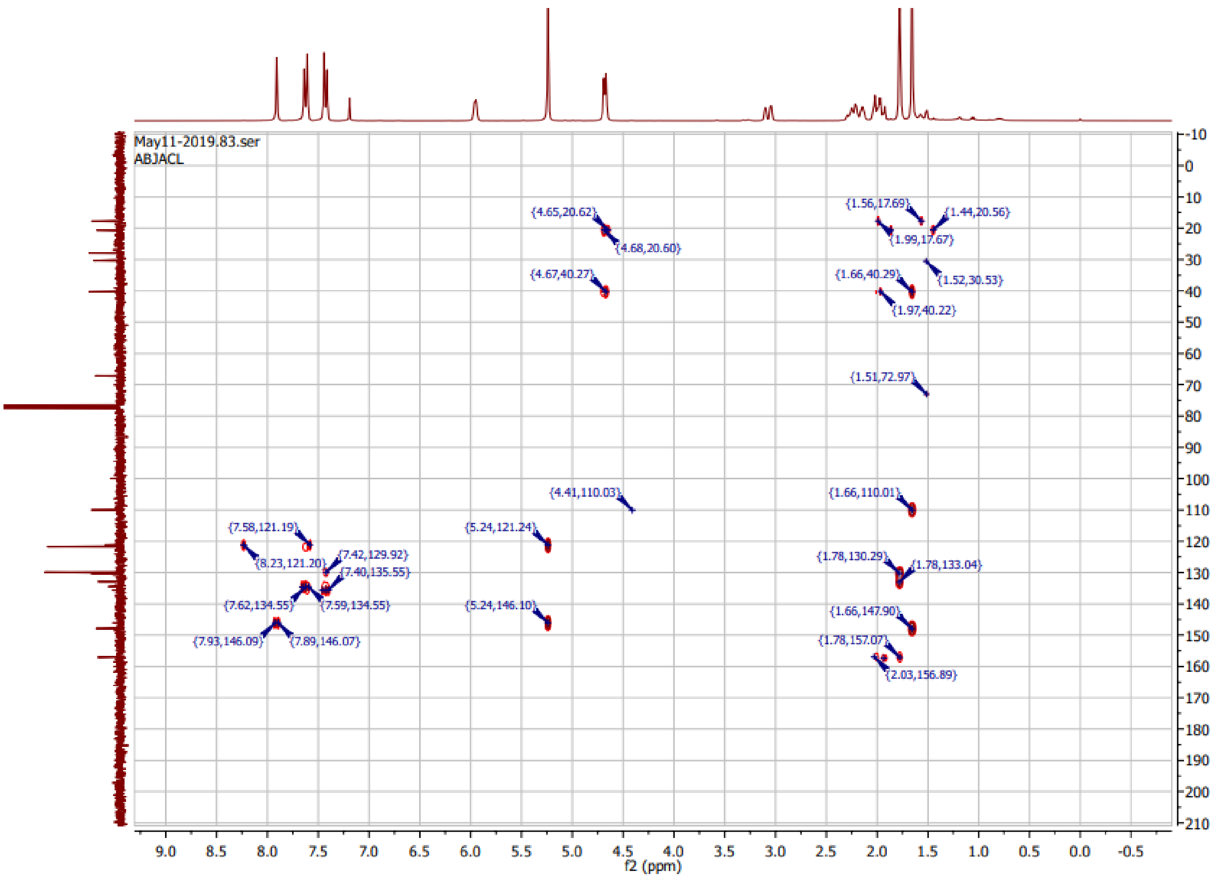

HMBC Spectrum

HRMS spectrum

Elemental Composition Report **Page 1**

Multiple Mass Analysis: 2 mass(es) processed
Tolerance = 5.0 PPM / DBE: min = -1.0, max = 16.0
Element prediction: Off
Number of isotope peaks used for i-FIT = 4

Monoisotopic Mass, Even Electron Ions
827 formula(e) evaluated with 4 results within limits (all results (up to 1000) for each mass)
Elements Used:
C: 0-500 H: 0-1000 N: 0-4 O: 0-4 35Cl: 0-1 37Cl: 0-1

AB123 1:9
11-Dec-2019
1912126 590 (3.561) Cm (590:591-(559:561+645:646)) 1: TOF MS ES+ 3.25e+004

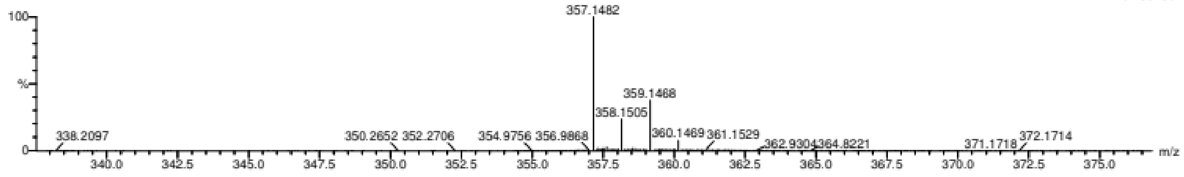

Minimum:	30.00				-1.0			
Maximum:	100.00		5.0	5.0	16.0			
Mass	RA	Calc. Mass	mDa	PPM	DBE	i-FIT	i-FIT (Norm)	Formula
357.1482	100.00	357.1482	0.0	0.0	10.5	565.5	0.6	C19 H22 N4 O 35Cl
		357.1491	-0.9	-2.5	14.5	565.7	0.8	C24 H21 O3
359.1468	37.39	359.1471	-0.3	-0.8	5.5	542.3	0.6	C18 H27 N2 O 35Cl 37Cl
		359.1453	1.5	4.2	10.5	542.4	0.8	C19 H22 N4 O 37Cl

1,2,3-triazole-Carvone 9d:

NMR Spectroscopy (500 MHz, $CDCl_3$)

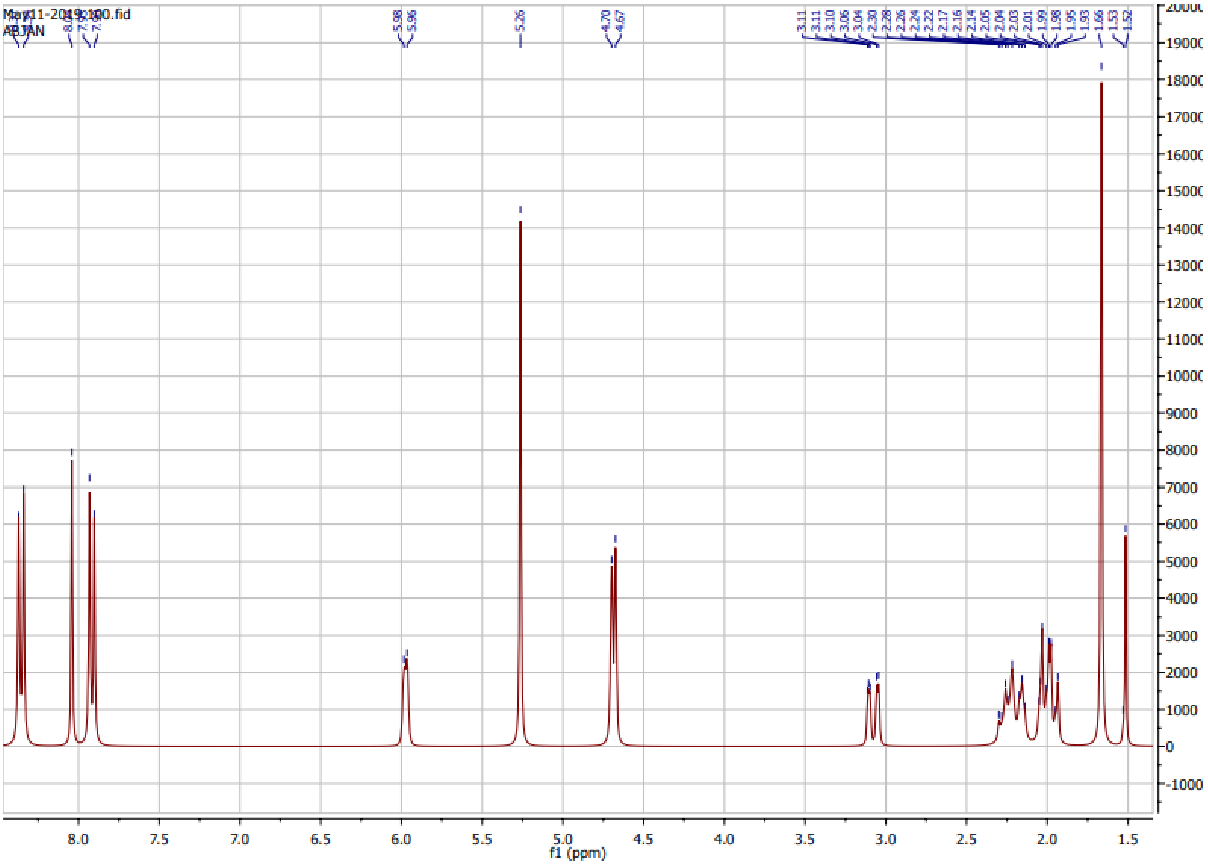

^{1}H NMR spectrum

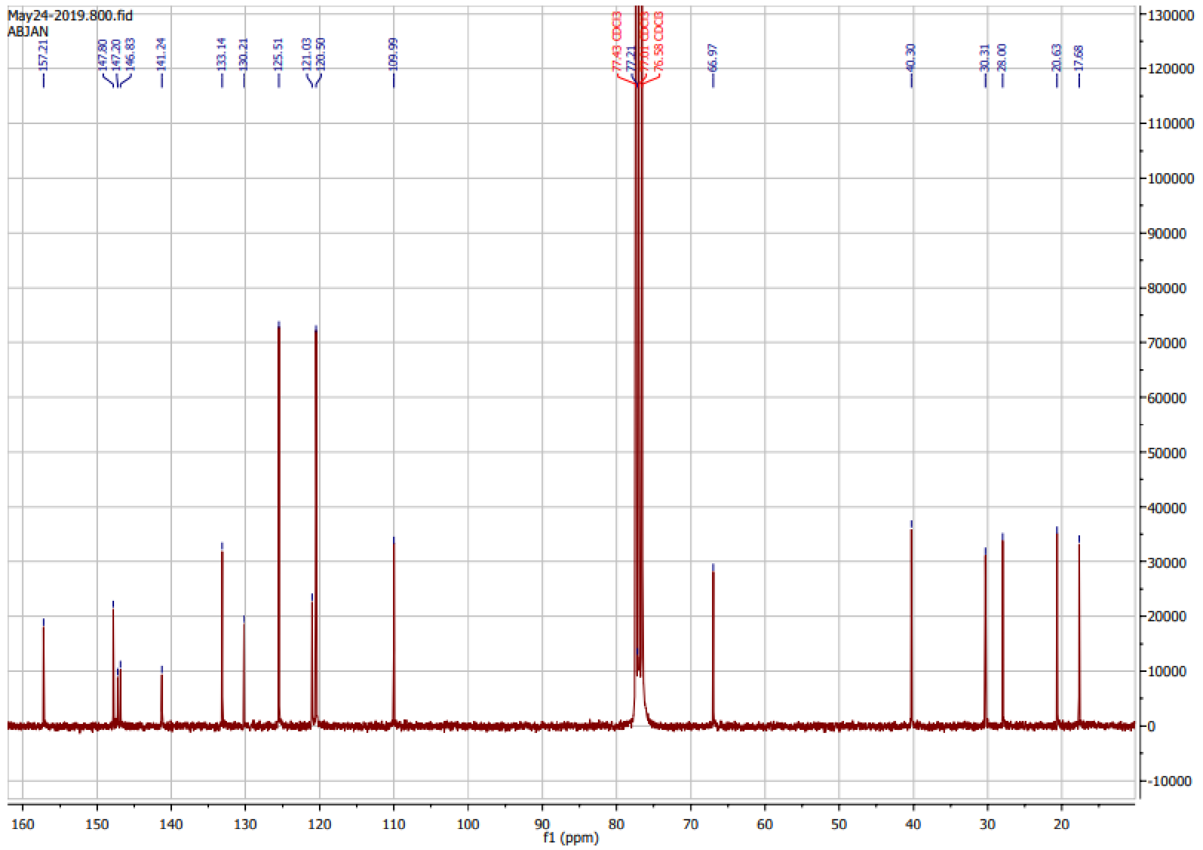

^{13}C Decoupled ^{1}H NMR spectrum

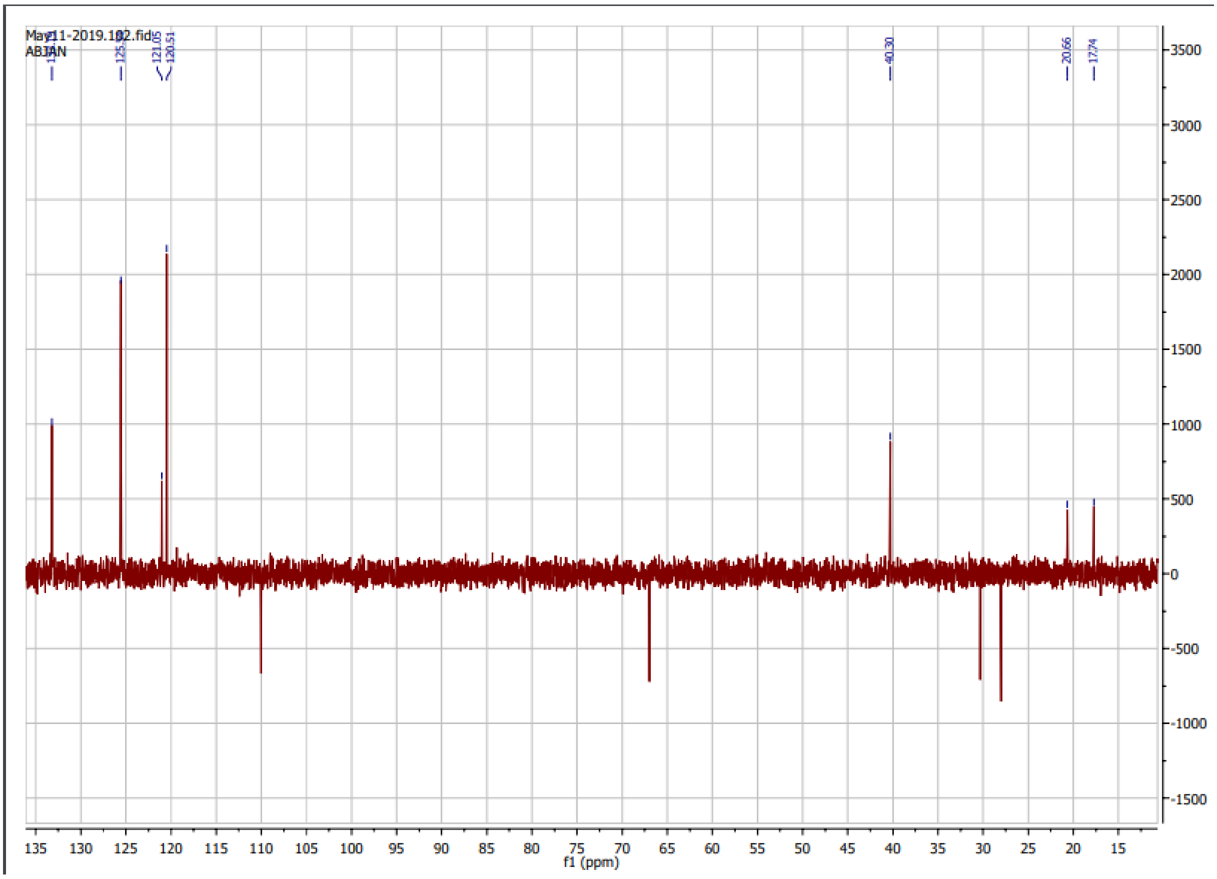

^{13}C Decoupled^{1}H NMR spectrum DEPT 135 Mode

HRMS spectrum

Elemental Composition Report **Page 1**

Single Mass Analysis
Tolerance = 5.0 PPM / DBE: min = -1.0, max = 16.0
Element prediction: Off
Number of isotope peaks used for i-FIT = 4

Monoisotopic Mass, Even Electron Ions
162 formula(e) evaluated with 1 results within limits (all results (up to 1000) for each mass)
Elements Used:
C: 0-500 H: 0-1000 N: 0-6 O: 0-4

AB124 1:10
11-Dec-2019
1912127 555 (3.362) Cm (552:555-(512:516+603:605)) 1: TOF MS ES+ 1.25e+004

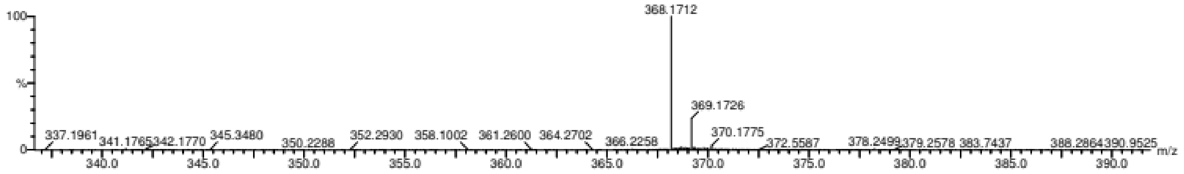

Minimum:				-1.0			
Maximum:		5.0	5.0	16.0			
Mass	Calc. Mass	mDa	PPM	DBE	i-FIT	i-FIT (Norm)	Formula
368.1712	368.1723	-1.1	-3.0	11.5	498.5	0.0	C19 H22 N5 O3

1,2,3-triazole-Carvone 9e:

NMR Spectroscopy (500 MHz, $CDCl_3$)

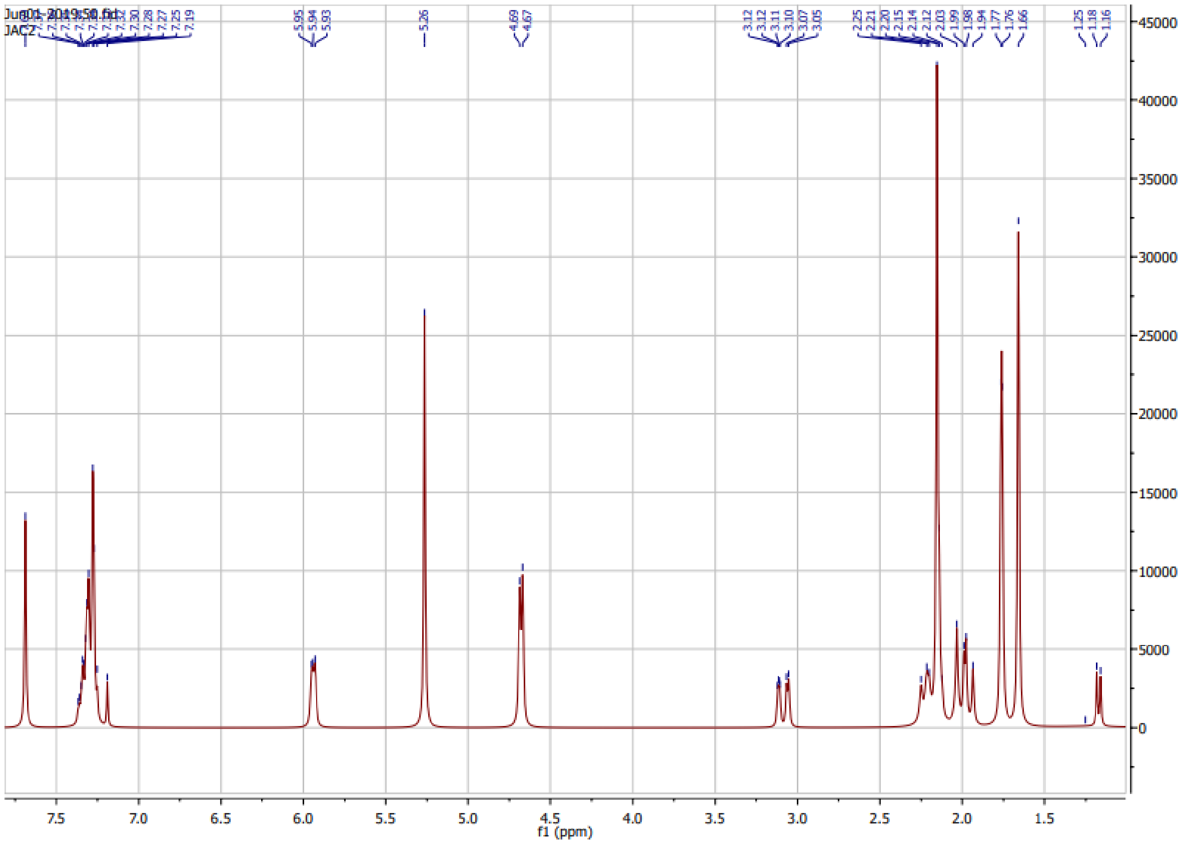

^{1}H NMR spectrum

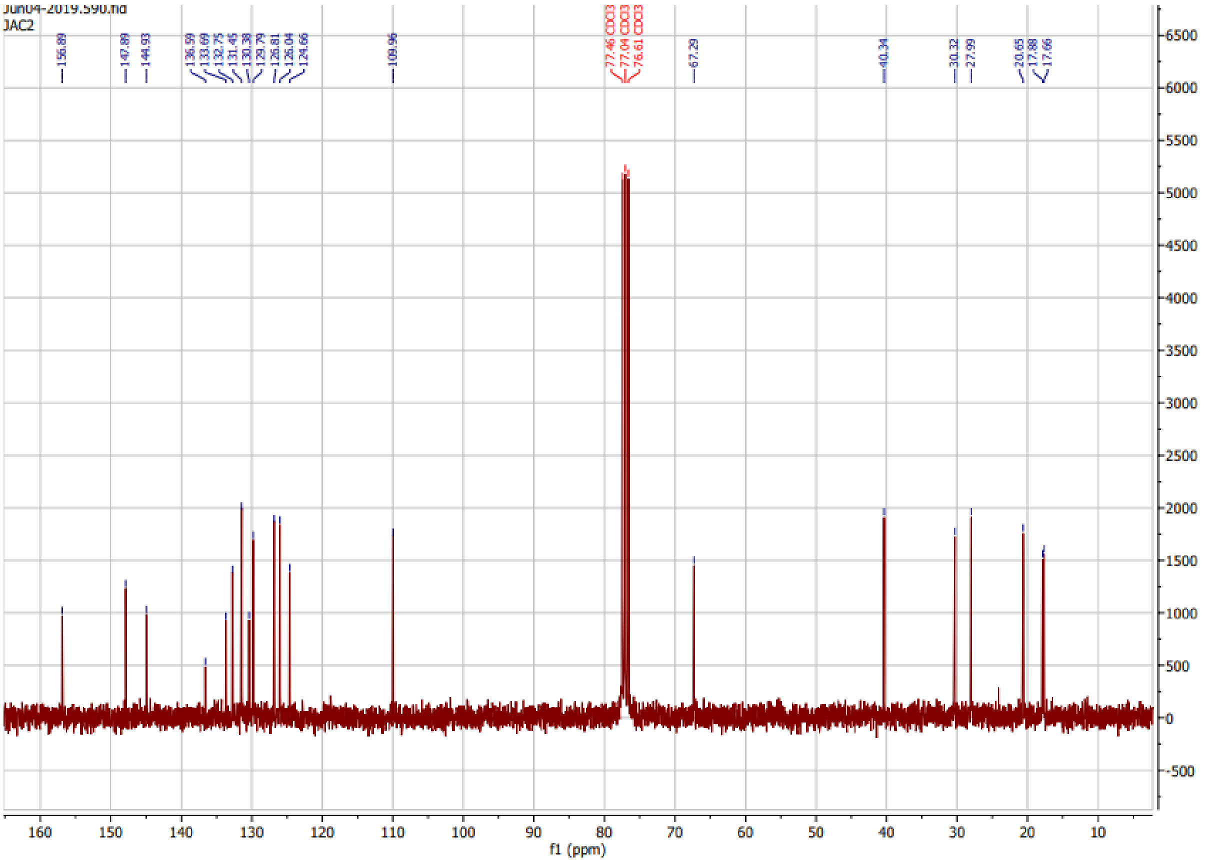

^{13}C Decoupled ^{1}H NMR spectrum

HRMS spectrum

Elemental Composition Report **Page 1**

Single Mass Analysis
Tolerance = 5.0 PPM / DBE: min = -1.0, max = 16.0
Element prediction: Off
Number of isotope peaks used for i-FIT = 4

Monoisotopic Mass, Even Electron Ions
97 formula(e) evaluated with 1 results within limits (all results (up to 1000) for each mass)
Elements Used:
C: 0-500 H: 0-1000 N: 0-6 O: 0-2

AB127 1:13

11-Dec-2019
1912130 571 (3.442) Cm (571:572-(536:537+618:619)) 1: TOF MS ES+ 6.12e+003

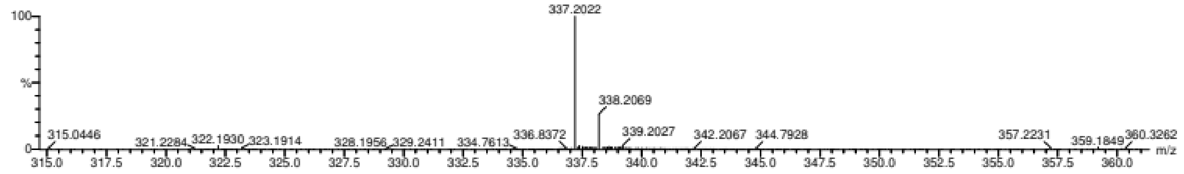

	Mass	Calc. Mass	mDa	PPM	DBE	i-FIT	i-FIT (Norm)	Formula
Minimum:					-1.0			
Maximum:			5.0	5.0	16.0			
	337.2022	337.2028	-0.6	-1.8	10.5	374.6	0.0	C20 H25 N4 O

1,2,3-triazole-Carvone 9f:

NMR Spectroscopy (500 MHz, $CDCl_3$)

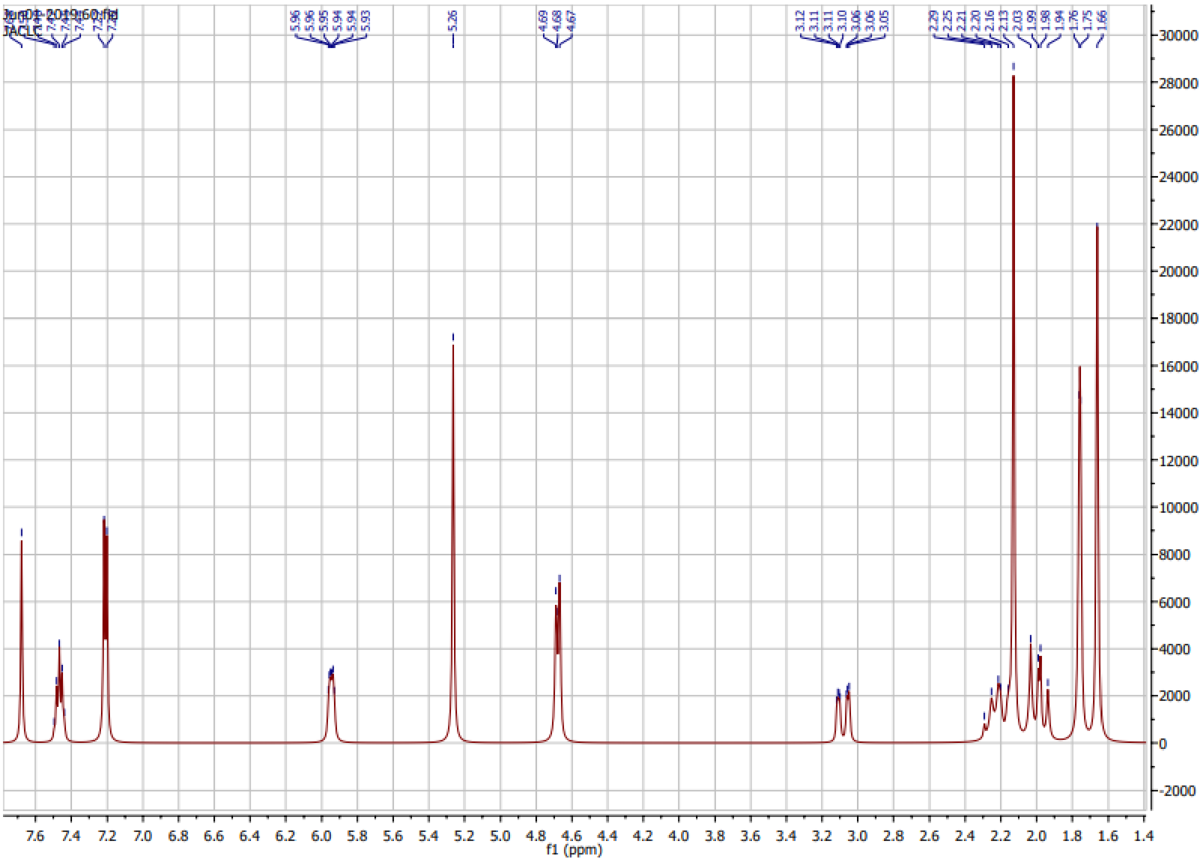

^{1}H NMR spectrum

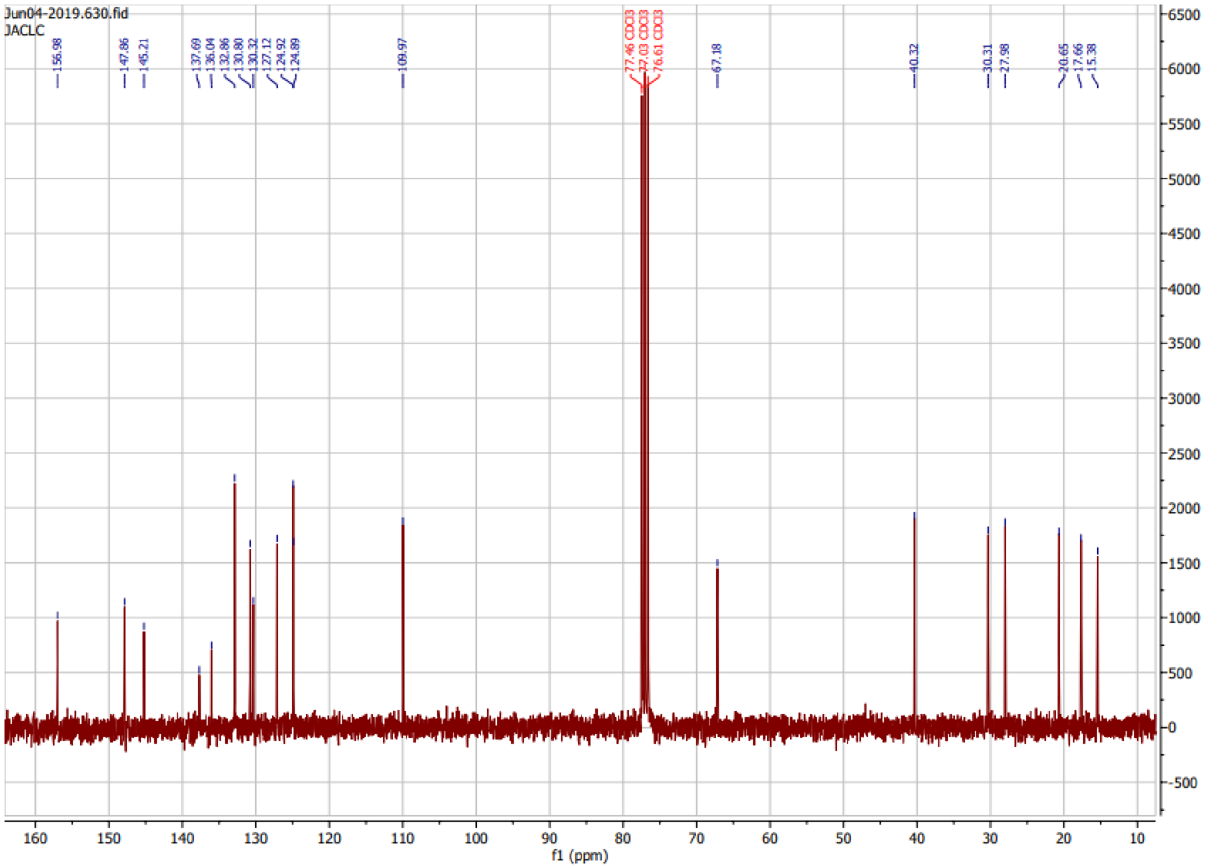

^{13}C Decoupled^{1}H NMR spectrum

HRMS spectrum

Elemental Composition Report

Multiple Mass Analysis: 2 mass(es) processed
Tolerance = 5.0 PPM / DBE: min = -1.0, max = 16.0
Element prediction: Off
Number of isotope peaks used for i-FIT = 4

Monoisotopic Mass, Even Electron Ions
739 formula(e) evaluated with 4 results within limits (all results (up to 1000) for each mass)
Elements Used:
C: 0-500 H: 0-1000 N: 0-6 O: 0-2 35Cl: 0-1 37Cl: 0-1

AB126 1:12

11-Dec-2019
1912129 605 (3.633) Cm (602:605-(571:573+665:667)) 1: TOF MS ES+ 1.36e+004

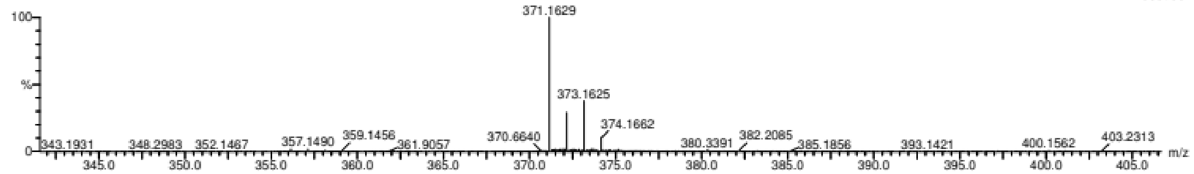

Mass	RA	Calc. Mass	mDa	PPM	DBE	i-FIT	i-FIT (Norm)	Formula
Minimum:	30.00				-1.0			
Maximum:	100.00		5.0	5.0	16.0			
371.1629	100.00	371.1620	0.9	2.4	15.5	541.0	0.4	C21 H19 N6 O
		371.1639	-1.0	-2.7	10.5	541.7	1.1	C20 H24 N4 O 35Cl
373.1625	37.60	373.1609	1.6	4.3	10.5	434.8	0.1	C20 H24 N4 O 37Cl
		373.1627	-0.2	-0.5	5.5	436.7	2.0	C19 H29 N2 O 35Cl 37Cl

1,2,3-triazole-Carvone 9g:

NMR Spectroscopy (500 MHz, $CDCl_3$)

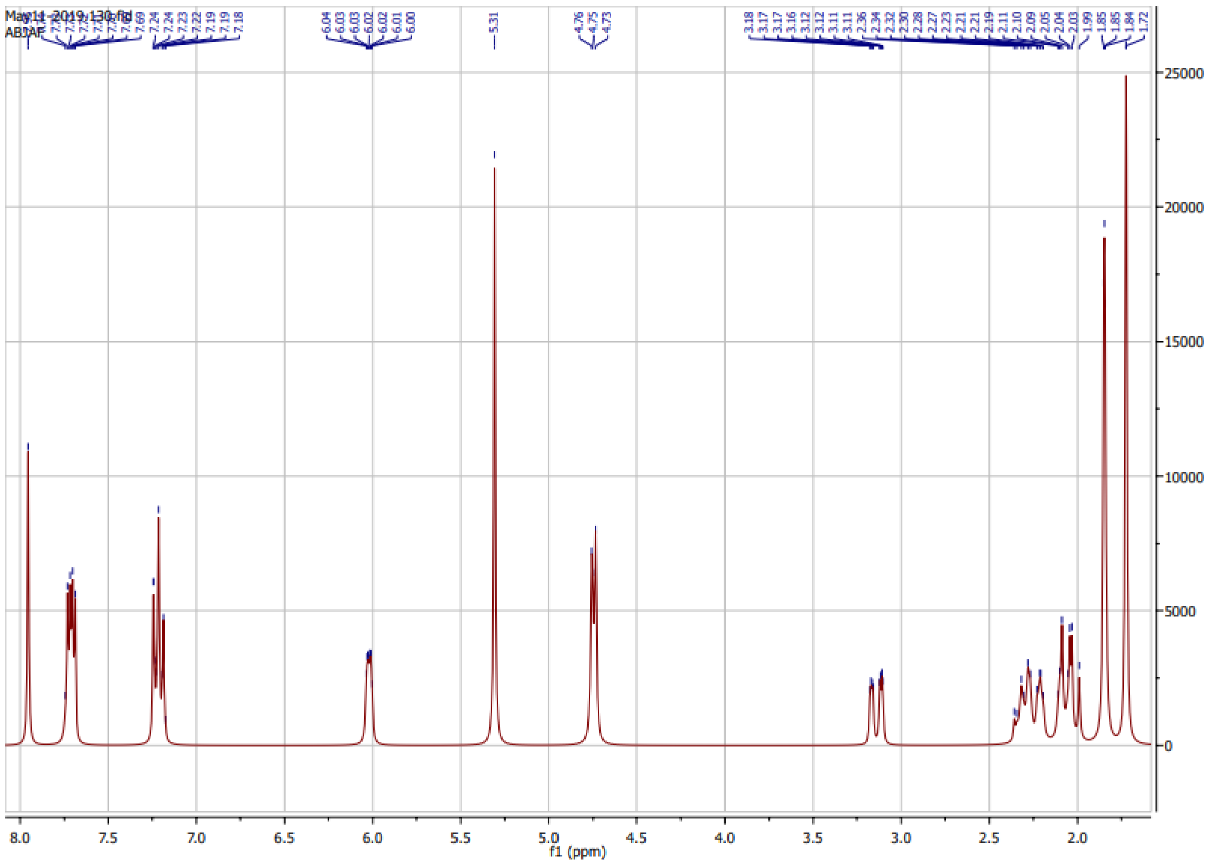

^{1}H NMR spectrum

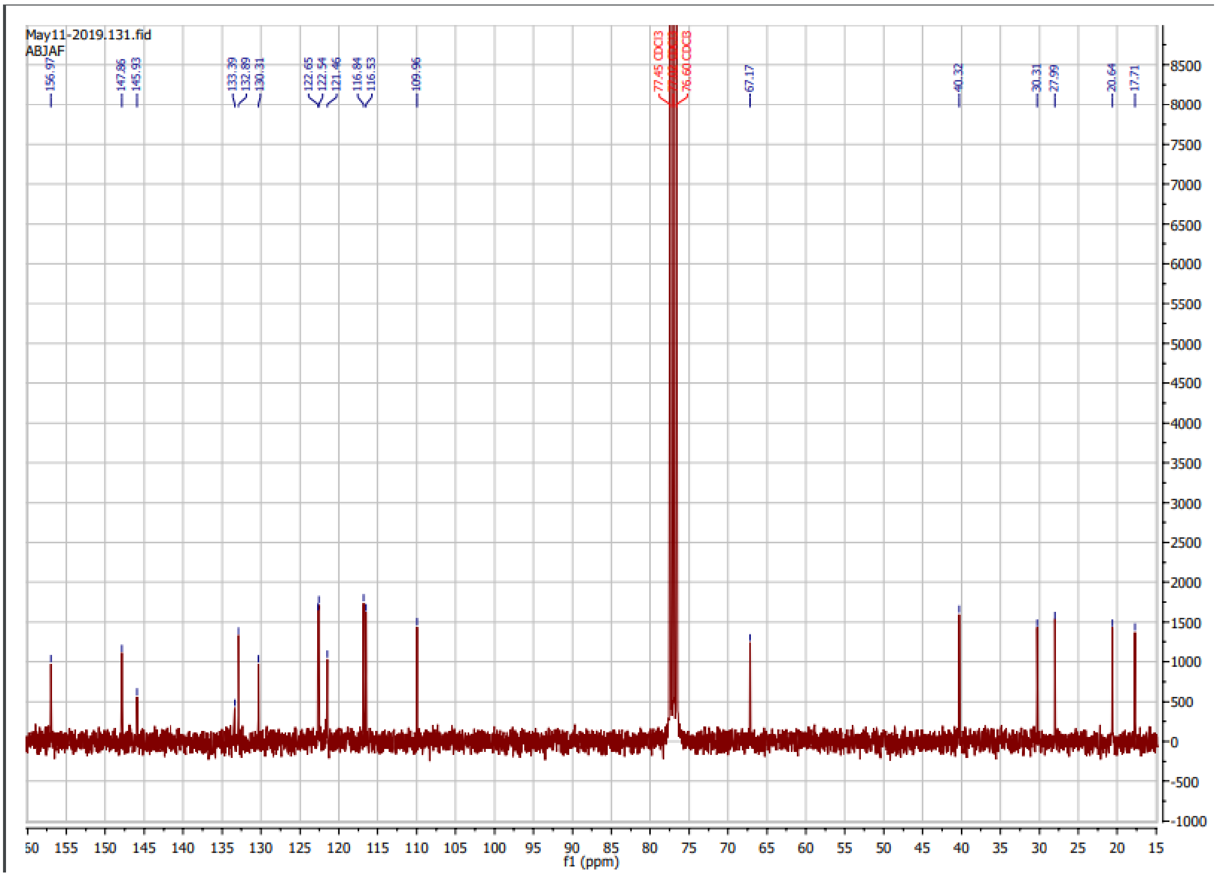

^{13}C Decoupled ^{1}H NMR spectrum

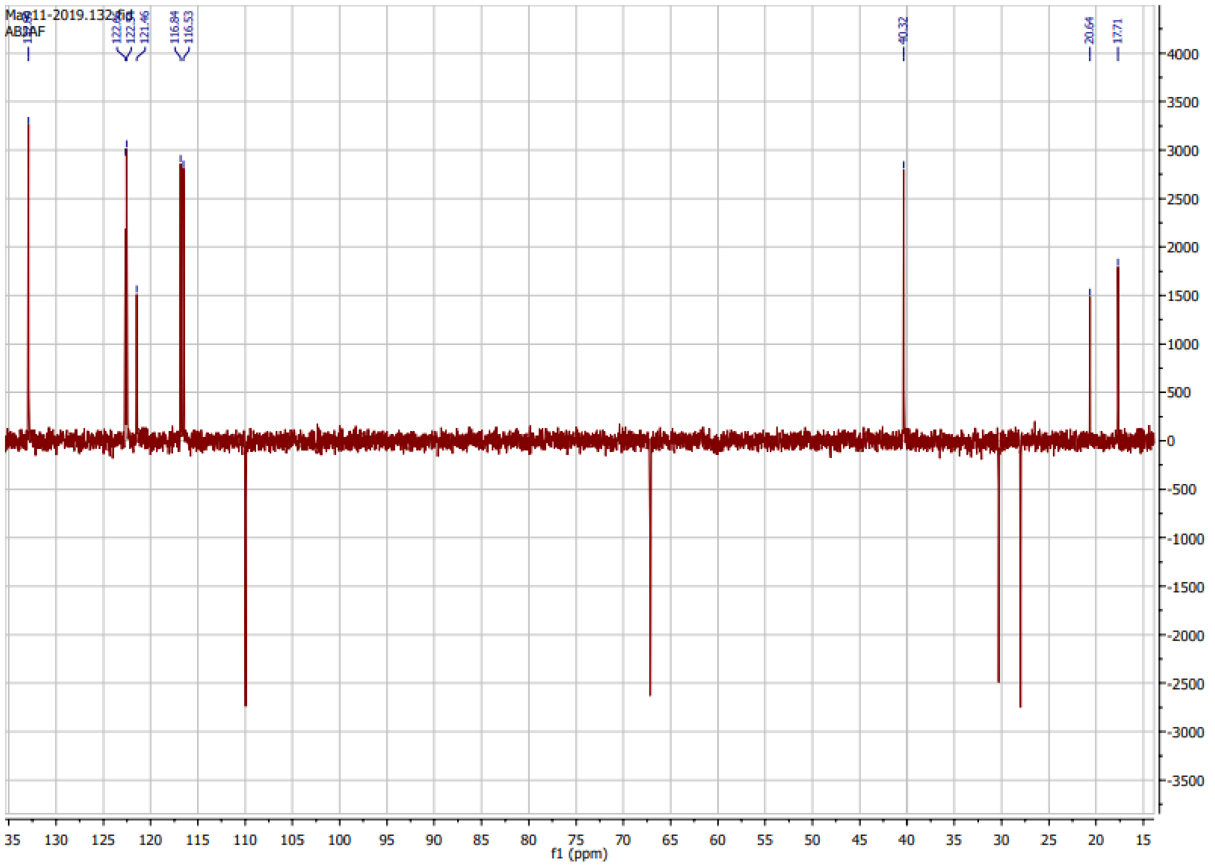

^{13}C Decoupled^{1}H NMR spectrum DEPT 135 Mode

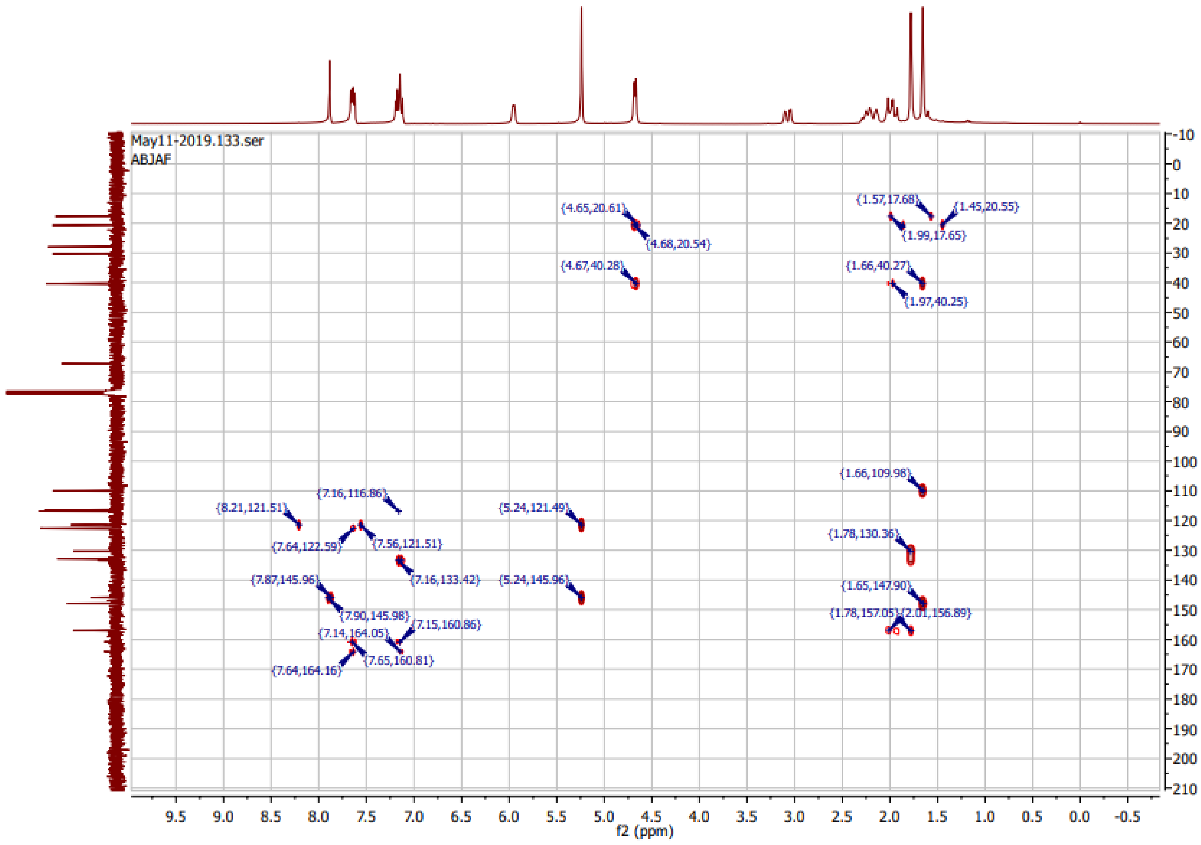

HMBC Spectrum

HRMS spectrum

Elemental Composition Report **Page 1**

Single Mass Analysis
Tolerance = 5.0 PPM / DBE: min = -1.0, max = 16.0
Element prediction: Off
Number of isotope peaks used for i-FIT = 4

Monoisotopic Mass, Even Electron Ions
295 formula(e) evaluated with 2 results within limits (all results (up to 1000) for each mass)
Elements Used:
C: 0-500 H: 0-1000 N: 0-6 O: 0-4 F: 0-1

AB125 1:11

11-Dec-2019
1912128 553 (3.357) Cm (552:558-(517:520+592:594)) 1: TOF MS ES+ 2.79e+004

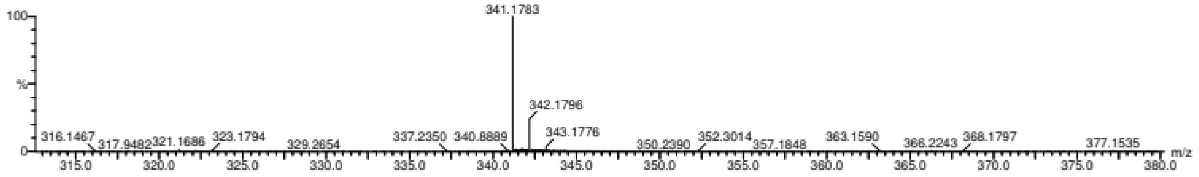

Minimum:				-1.0			
Maximum:		5.0	5.0	16.0			
Mass	Calc. Mass	mDa	PPM	DBE	i-FIT	i-FIT (Norm)	Formula
341.1783	341.1778	0.5	1.5	10.5	698.3	0.6	C19 H22 N4 O F
	341.1766	1.7	5.0	14.5	698.6	0.8	C22 H21 N4

1,2,3-triazole-Carvone 9h:

NMR Spectroscopy (500 MHz, $CDCl_3$)

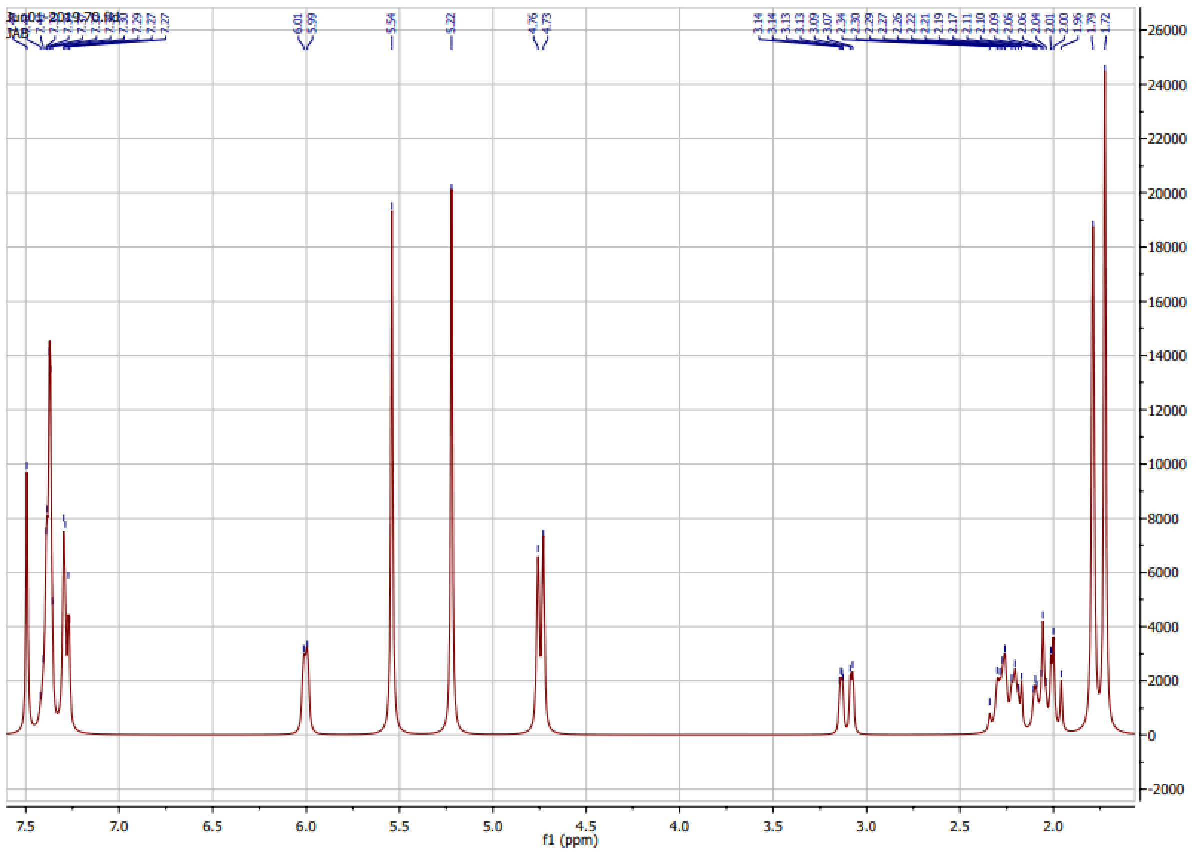

^{1}H NMR spectrum

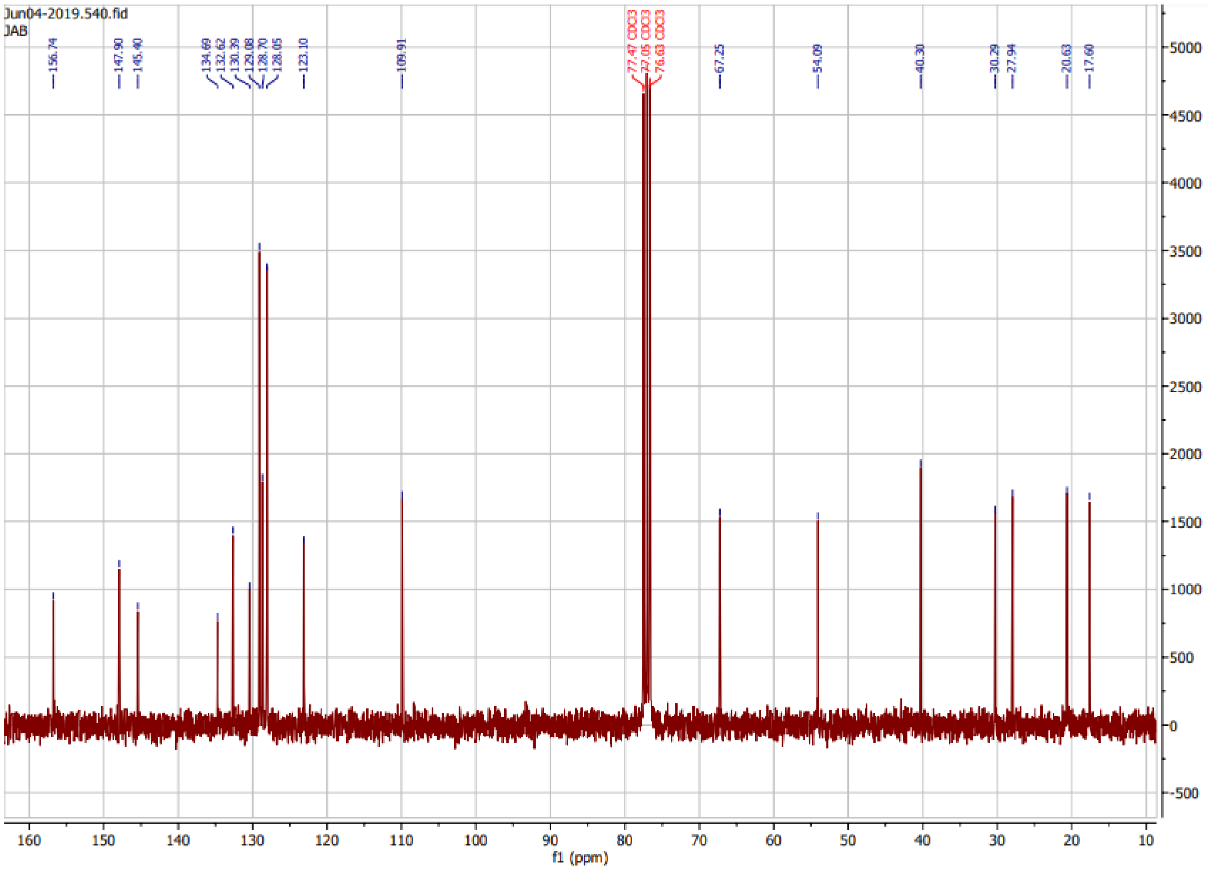

^{13}C Decoupled^{1}H NMR spectrum

HRMS spectrum

Elemental Composition Report **Page 1**

Single Mass Analysis
Tolerance = 5.0 PPM / DBE: min = -1.0, max = 16.0
Element prediction: Off
Number of isotope peaks used for i-FIT = 4

Monoisotopic Mass, Even Electron Ions
97 formula(e) evaluated with 1 results within limits (all results (up to 1000) for each mass)
Elements Used:
C: 0-500 H: 0-1000 N: 0-6 O: 0-2

AB128 1:14

11-Dec-2019
1912131 527 (3.199) Cm (525:531-(486:493+609:613)) 1: TOF MS ES+ 1.06e+005

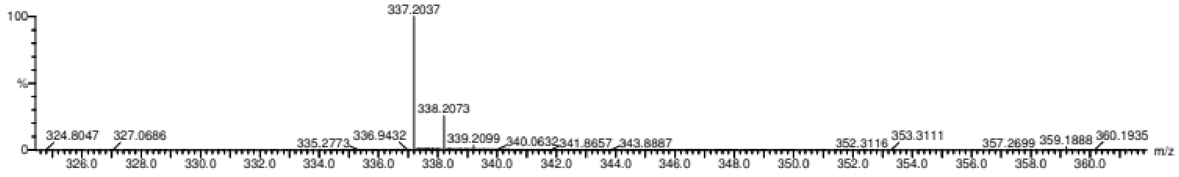

Minimum:				-1.0			
Maximum:		5.0	5.0	16.0			
Mass	Calc. Mass	mDa	PPM	DBE	i-FIT	i-FIT (Norm)	Formula
337.2037	337.2028	0.9	2.7	10.5	874.2	0.0	C20 H25 N4 O

II- Calculated Supporting Information

Table S1. The calculated energies (**E**. a.u). zero-point vibrational energies (**ZPE**. a.u). thermal corrections (**TCE**. a.u). entropy values (**S**. cal/mol/K). ZPE corrected energies (**Ecorr**. a.u). enthalpies (**H**. a.u). and **TΔS** (a.u). at 25°C. for all possible regioisomers of the 32CA between **7** and **8a**.

	E	ZPE	TCE	S	Ecorr	H	TΔS
**7**	-635.43563	0.27064	0.28775	130.072	-635.17024	-635.15313	
**8a**	-395.83829	0.10381	0.11174	83.866	-395.73649	-395.72857	
**TS-1**	-1031.24700	0.37528	0.40009	173.479	-1030.87900	-1030.85419	-0.01922305
**TS-2**	-1031.24806	0.37579	0.40032	171.931	-1030.87956	-1030.85503	-0.04058227
**9a-1**	-1031.38420	0.38224	0.40555	166.487	-1031.00937	-1030.98606	-0.08501066
**9a-2**	-1031.38213	0.38291	0.40591	164.796	-1031.00665	-1030.98365	-0.08249203
**TS-3**	-1031.21294	0.37511	0.39945	166.017	-1030.84511	-1030.82077	-0.07852189
**TS-4**	-1031.22323	0.37575	0.39994	165.163	-1030.85477	-1030.83057	-0.07870434
**9a-3**	-1031.25331	0.37819	0.40207	162.967	-1030.88245	-1030.85857	-0.07992208
**9a-4**	-1031.25614	0.37885	0.40262	162.891	-1030.88465	-1030.86088	-0.07850906
**TS-5**	-1031.24122	0.37561	0.40012	167.563	-1030.87289	-1030.84839	-0.0752098
**TS-6**	-1031.23907	0.37590	0.40029	167.439	-1030.87046	-1030.84608	-0.07745239
**9a-5**	-1031.31033	0.38054	0.40425	165.092	-1030.93717	-1030.91346	-0.08072837
**9a-6**	-1031.29802	0.38035	0.40392	161.977	-1030.92504	-1030.90147	-0.08103435
**TS-7**	-1031.23639	0.37507	0.39980	171.549	-1030.86858	-1030.84386	-0.07389133
**TS-8**	-1031.23972	0.37537	0.40000	169.726	-1030.87163	-1030.84700	-0.07782536
**9a-7**	-1031.29455	0.37978	0.40352	163.765	-1030.92214	-1030.89840	-0.08433931
**9a-8**	-1031.28996	0.38019	0.40373	162.19	-1030.91714	-1030.89360	-0.08138927

Table S2. Energies (**ΔE**). enthalpies (**ΔH**) and Gibbs free energies (**ΔG**) for stationary points of all possible regioisomers of the 32CA between **7** and **8a** relative to the separate reactants. All values are in kcal/mol.

	**ΔE**	**ΔH**	**ΔG**
**TS-1**	17.407	17.265	17.284
**TS-2**	17.053	16.739	16.780
**9a-1**	-64.408	-65.490	-65.405
**9a-2**	-62.702	-63.976	-63.893
**TS-3**	38.668	38.233	38.312
**TS-4**	32.611	32.084	32.163
**9a-3**	15.238	14.515	14.595
**9a-4**	13.860	13.068	13.147
**TS-5**	21.238	20.906	20.981
**TS-6**	22.763	22.355	22.433
**9a-5**	-19.100	-19.931	-19.850
**9a-6**	-11.488	-12.406	-12.325
**TS-7**	23.940	23.746	23.820
**TS-8**	22.026	21.773	21.851
**9a-7**	-9.666	-10.479	-10.394
**9a-8**	-6.530	-7.469	-7.387

Table S3. The calculated energies (**E**. a.u). zero-point vibrational energies (**ZPE**. a.u). thermal corrections (**TCE**. a.u). entropy values (**S**. cal/mol/K). ZPE corrected energies (**Ecorr**. a.u). enthalpies (**H**. a.u). and **TΔS** (a.u). at 25°C. for all possible regioisomers of the 32CA between **2Cu(I)-7** and **8a**.

	E	ZPE	TCE	S	Ecorr	H	TΔS
**8a**	-395.838289	0.103814	0.111737	83.867	-395.736489	-395.728566	
**2Cu(I)-7**	-4225.412542	0.430132	0.460296	199.455	-4224.99076	-4224.96059	
**1.4-RC**	-4621.27622	0.534827	0.57387	246.549	-4620.75177	-4620.71272	-0.01747175
**1.4-TS1**	-4621.26758	0.534912	0.572319	234.747	-4620.74305	-4620.70564	-0.10037333
**1.4-In**	-4621.29405	0.537219	0.574408	233.133	-4620.76726	-4620.73007	-0.11790827
**1.4-TS2**	-4621.27158	0.536125	0.573163	232.73	-4620.74586	-4620.70882	-0.11172548
**1.4-P**	-4621.34718	0.539105	0.576523	239.273	-4620.81854	-4620.78112	-0.10765841
**1.5-RC**	-4621.27893	0.533908	0.57343	254.087	-4620.75538	-4620.71586	0.00703849
**1.5-TS1**	-4621.23078	0.533744	0.572235	247.335	-4620.70739	-4620.6689	-0.00320804
**1.5-In**	-4621.28796	0.536903	0.574561	237.412	-4620.76147	-4620.72381	-0.12543757
**1.5-TS2**	-4621.28313	0.536343	0.573377	234.29	-4620.75719	-4620.72016	-0.11899821
**1.5-P**	-4621.33895	0.538707	0.576139	238.625	-4620.81069	-4620.77326	-0.11074055

Table S4. Energies (**E**. a.u), zero-point vibrational energies (**ZPE**. a.u), thermal corrections (**TCE**. a.u), entropy values (**S**. cal/mol/K), ZPE corrected energies (**Ecorr**. a.u), enthalpies (**H**. a.u) and **TΔS** (a.u) at 25°C calculated at B3LYP/6-31G* in ethanol as solvent using the PCM model for the stationary points of the two regioisomers 1,4 and 1,5-disubstituted 1,2,3-triazole of the 32CA between **2Cu(I)-7** and **8a**.

	**E**	**ZPE**	**TCE**	**S**	**Ecorr**	**H**	**TΔS**
**8a**	-395.8419542	0.103804	0.111716	83.789	-395.740164	-395.732252	
**2Cu(I)-7**	-4225.472218	0.429337	0.458749	192.891	-4225.05121	-4225.0218	
**1.4-RC**	-4621.331064	0.533722	0.573069	253.554	-4620.8077	-4620.76835	-0.01098772
**1.4-TS1**	-4621.324008	0.533819	0.570615	231.42	-4620.80054	-4620.76375	-0.1021636
**1.4-In**	-4621.361224	0.536504	0.572105	221.902	-4620.83513	-4620.79953	-0.1249919
**1.4-TS2**	-4621.337476	0.535647	0.571819	226.536	-4620.81222	-4620.77605	-0.10775154
**1.4-P**	-4621.415718	0.538801	0.576204	236.829	-4620.88737	-4620.84997	-0.10054058
**1.5-RC**	-4621.336682	0.533083	0.571901	253.166	-4620.81394	-4620.77512	-0.01117207
**1.5-TS1**	-4621.299033	0.532856	0.569734	235.308	-4620.77651	-4620.73964	-0.10031632
**1.5-In**	-4621.349428	0.536167	0.574034	240.744	-4620.82366	-4620.7858	-0.11603961
**1.5-TS2**	-4621.342388	0.535526	0.570044	216.541	-4620.81725	-4620.78273	-0.11250041
**1.5-P**	-4621.403118	0.538063	0.573786	225.281	-4620.87549	-4620.83977	-0.10602731
